# Rbfox1 regulates alternative splicing of *Nrcam* in primary sensory neurons to mediate peripheral nerve injury-induced neuropathic pain

**DOI:** 10.1016/j.neurot.2023.e00309

**Published:** 2023-12-19

**Authors:** Long He, Haoyu Guo, Hongwei Wang, Kuicheng Zhu, Da Li, Chaofan Zhang, Yanqiu Ai, Jian-jun Yang

**Affiliations:** a Department of Anesthesiology, Pain and Perioperative Medicine, The First Affiliated Hospital of Zhengzhou University, Zhengzhou 450000, China; b Department of Laboratory Animal Resources, Academy of Medical Sciences, Zhengzhou University, Zhengzhou 450000, China

**Keywords:** RNA-Binding Fox1, Alternative splicing, Neuron-glial related cell adhesion molecule, Neuropathic pain, Primary sensory neurons

## Abstract

The primary sensory neurons of the dorsal root ganglia (DRG) are subject to transcriptional alterations following peripheral nerve injury. These alterations are believed to play a pivotal role in the genesis of neuropathic pain. Alternative RNA splicing is a process that generates multiple transcript variants from a single gene, significantly contributing to the complexity of the transcriptome. However, little is known about the functional significance and control of alternative RNA splicing in injured DRG after spinal nerve ligation (SNL). In our study, we conducted a comprehensive transcriptome profiling and bioinformatic analysis to approach and identified a neuron-specific isoform of an RNA splicing regulator, RNA-binding Fox1 (Rbfox1, also known as A2BP1), as a crucial regulator of alternative RNA splicing in injured DRG after SNL. Notably, Rbfox1 expression is markedly reduced in injured DRG following peripheral nerve injury. Restoring this reduction effectively mitigates nociceptive hypersensitivity. Conversely, mimicking the downregulation of Rbfox1 expression generates neuropathic pain symptoms. Mechanistically, we uncovered that Rbfox1 may be a key factor influencing alternative RNA splicing of neuron-glial related cell adhesion molecule (NrCAM), a key neuronal cell adhesion molecule. In injured DRG after SNL, the downregulation of Rbfox1amplifies the insertion of exon 10 in *Nrcam* transcripts, leading to an increase in long *Nrcam* variants (L-*Nrcam*) and a corresponding decrease in short *Nrcam* variants (S-*Nrcam*) within injured DRG. In summary, our study supports the essential role of Rbfox1 in neuropathic pain within DRG, probably via the regulation of *Nrcam* splicing. These findings suggest that Rbfox1 could be a potential target for neuropathic pain therapy.

## Introduction

Neuropathic pain, a prevalent clinical issue resulting from peripheral nerve injury, is characterized by heightened sensitivity to painful stimulation (hyperalgesia), pain triggered by normally innocuous stimuli (allodynia), and spontaneous ongoing pain [1]. Existing medications often provide limited and nonspecific relief for neuropathic pain patients, as they do not specifically target the underlying causes of neuropathic pain. Peripheral nerve injuries lead to maladaptive changes in the expression of pain-associated genes in injured sensory neurons and throughout the central nervous system's nociceptive pathways, contributing to the genesis of neuropathic pain [[2], [3], [4]]. While multiple cellular and molecular pathways are likely involved in these changes, the exact mechanisms remain poorly understood. A deeper understanding of these changes in the dorsal root ganglia (DRG) following peripheral nerve injury could pave the way for novel, neuropathic pain-specific therapies.

Alternative RNA splicing is a widespread post-transcriptional process in multicellular eukaryotes, generating multiple mature transcripts from a single gene. This process significantly contributes to the complexity of the transcriptome and is essential for refining cellular identity and function [5,6]. The regulation of alternative RNA splicing is regulated by *cis*-regulatory enhancers and silencers located within pre-mRNAs, which interact with *trans*-acting splicing factors. Dysregulated alternative RNA splicing plays a significant role in several human diseases, including neuropathic pain, genetic disorders, cognitive function, and addiction [[7], [8], [9], [10]]. Notably, disruption of alternatively spliced isoform levels, rather than disruption of gene expression levels, are a primary source of pathological effects in psychiatric and neurological diseases [[11], [12], [13]].

Alternative RNA splicing is regulated by RNA-binding proteins (RBPs) [14]. Dysfunction in neural RBPs has been associated with neurological disease [15]. Several RBPs such as serine-arginine splice factor 1 (SRSF1), have been shown to control pathological neuropathic pain by regulating the splicing of genes like vascular endothelial growth factor A (VEGF-A) [16]. Evidence also suggests that alternative RNA splicing variations in ion channels and receptors play a role in nociception and chronic pain. For example, *Cacna1b*, which encodes the voltage-gated Ca_v_2.2 functional core 1 subunit, and cell-specific AS of Cacna1b are involved in intrathecal morphine analgesia, but it is disturbed by peripheral nerve injury, which contributes to the development of neuropathic pain [17,18]. In addition, Na_v_1.7 is predominantly expressed in small and medium-sized DRG neurons, the majority of which are nociceptive. Na_v_1.7 splicing variations are related to neuropathy and congenital insensitivity to pain, while Nav1.7 variants have been identified in DRGs under scenarios of neuropathic pain [19,20]. However, the underlying molecular pathways and contributions of alternative RNA splicing to the pathogenesis of neuropathic pain require further investigation.

In our study, we conducted a global analysis of alternative RNA splicing in injured DRG following spinal nerve ligation (SNL). We identified Rbfox1 (also known as A2BP1) as a key *trans*-acting RNA splicing regulator in injured DRG neurons following SNL, Rbfox1 is highly expressed in the brain, cardiac muscle, and skeletal muscle, and it has been implicated in various neurodevelopmental and neuropsychiatric conditions, including epilepsy, intellectual disability, autism, and schizophrenia [[21], [22], [23]]. Additionally, Rbfox1 plays a crucial role in cardiac and skeletal muscle development and function. It regulates the splicing of genes important for synapse assembly (NRXN3, NrCAM) and synaptic transmission (Syntaxin 3, STX3, SNAP25) [24,25]. Moreover, Rbfox1 targets ion channels and neurotransmitter receptors, serving as a key regulator of genes involved in neuronal excitability and network function. Such as *SCN3A* (encoding Na_v_ 1.3), *SCN8A* (encoding Na_v_ 1.6), *Grin1* (encoding N-methyl D-aspartate receptor 1), and *Atp2b1* (encoding calcium ATPase) [24,26,27].

In our study, we found that the downregulation of Rbfox1 in injured DRG significantly influences the development of nociceptive hypersensitivity following SNL. Rescuing Rbfox1 expression mitigated pain sensitivity while mimicking this downregulation in normal mice produced pain symptoms. At a mechanistic level, we discovered that Rbfox1 directly regulates *Nrcam* exon 10 splicing via exon skipping. Rbfox1-mediated upregulation of the long *Nrcam* RNA variant (including exon 10) in injured DRG eventually contributes to the genesis of neuropathic pain. Consequently, the downregulation of DRG Rbfox1 is likely crucial for peripheral nerve injury–induced nociceptive hypersensitivity.

## Materials and methods

### Animals

Male and female C57BL/6 mice weighing 25–30 ​g were used for the experiments. The mice were acquired from Charles River Laboratories (Beijing, China). All mice were housed centrally at Zhengzhou University under a conventional 12-h light/dark cycle, with ad libitum access to water and food pellets. The Medical Experimental Animal Administrative Committee of Zhengzhou University approved all experimental protocols, which conformed with the ethical guidelines of the International Association for the Study of Pain. Animals were numbered, allocated randomly to several experimental groups using a random number table, and tested in sequence. Following experimentation, the mice were euthanized via inhalation of sevoflurane. Experimenters were blinded to the treatment conditions performed testing and biochemical studies. All mice were habituated for 1–2 ​h daily for 2–3 days before a test of the baseline behavior. All studies were performed between 8:00 a.m. and 8:00 p.m. Every effort was made to minimize the number of animals utilized and their suffering.

### Neuropathic pain models

As described in a previously published study, the SNL-induced neuropathic pain model in mice was established [28,29]. Brieﬂy, the experimental mice were sevoflurane-anesthetized (inhaled concentration 1.5–2%) and placed in a prone position. On the lower back, a dorsolateral incision was created in the skin. After dissecting the surrounding tissues, the L_4_ transverse process was identified and removed unilaterally. Under a surgical microscope, the L_4_ spinal nerve was carefully isolated, ligated with a 6-0 silk suture, and then transected just distal to the ligature. The skin and muscles were layered and closed. The same procedure was performed on sham animals without transection and ligation of the L_4_ spinal nerve.

As previously described, the chronic constriction injury (CCI)-induced neuropathic pain model in mice was generated [30,31]. In brief, when mice were anesthetized as detailed before, the left sciatic nerve trunk was dissected well above the hip, and three ligatures were attached loosely with 6-0 silk thread approximately 2 ​mm apart proximal to the nerve's branching. When slight flicking of the ipsilateral hind limb was observed, the ligatures were loosened. The sham groups underwent similar procedures, but the sciatic nerve was not ligated.

### Behavioral test

Behavioral testing, including mechanical, heat, and cold tests, was conducted at 0.5–1 ​h intervals as described previously [28,30]. The conditioned place preference (CPP) test was conducted six weeks following viral microinjection, as reported previously [28]. After the behavioral tests described below were completed, locomotion functional testing was conducted as described.

For the mechanical test, each mouse was acclimated for 30 ​min in a Plexiglas chamber with an elevated mesh screen. Two calibrated von Frey filaments (0.07 and 0.4 ​g, Stoelting Co., USA) were utilized to stimulate the hind paw for 1–2 ​s and each hind paw 10 times at 5-min intervals. In each of these 10 trials, the occurrence of paw withdrawal was recorded as a percent response frequency [(number of paw withdrawals/10 trials) 100 ​= ​% response frequencies].

For the heat test, paw withdrawal latencies in response to noxious heat stimuli were recorded using an IITC Plantar Analgesia system (IITC Life Science Inc., LA, USA). Briefly, the mice were placed into individual Plexiglas cubes (8.5 ​cm long ​× ​5 ​cm wide ​× ​6 ​cm high) on the surface of the glass platform. The heat source was released through a hole focused on the central plantar surface of the hind paw. To prevent tissue injury, the heat source was switched off when the hind paw moved or after 20 ​s. Then, the duration from the heat source initiation to shut off was recorded as the paw withdrawal latency. A modification was made to the radiant heat intensity to achieve a basal paw withdrawal duration of 10–12 ​s. Each animal was tested 5 times at 5-min intervals on each side. To avoid tissue injury to the hind paw, a cutoff time of 20 ​s was used.

For the cold test, each mouse was placed in a Plexiglas chamber on a cold aluminum plate (0 ​°C). A digital thermometer was used to continuously monitor the temperature. Paw withdrawal latency was defined as the interval between the placement of the mouse and a positive nociceptive response (e.g., lifting the relevant paw or leaping). Each experiment was performed three times at intervals of 10 ​min. A 20-s threshold was utilized to prevent tissue injury to the hind paw.

For the CPP test, a device (Med Associates Inc., USA) with two Plexiglas chambers joined by an interior door was utilized. Each chamber had a distinctive floor texture and wall design. Photobeam detectors installed on the chamber walls monitored the mice's movement and time spent in each chamber, and MED-PC IV CPP software automatically collected these data. Mice were given 30 ​min of total access to both test chambers to familiarize themselves with the experimental environment. To evaluate whether mice had a preexisting chamber bias, the basal length of time spent in each chamber was measured within 15 ​min after preconditioning. Mice that spent more than 80% or less than 20% of their overall time in a certain chamber were eliminated from further testing. When the interior door was shut for three days, the following conditioning routine was carried out daily. The mice received an intrathecal injection of saline (5 ​μl) paired with one conditioning chamber for 15 ​min in the morning. Six hours later, lidocaine (0.8 ​% in 5 ​μl of saline) was administered intrathecally and paired with another conditioning chamber for 15 ​min in the afternoon. Every day, the injection sequence of saline and lidocaine was switched. On the day of the experiment, the mouse was randomly placed in one of the open-doored chambers. For 15 ​min, the movement and length of time spent in each chamber by each mouse were recorded to determine chamber preference. Subtracting the preconditioning time from the test duration in the lidocaine chamber was used to determine the difference in scores.

Mouse locomotor activity tests included placing, grasping, and righting reflexes. Placing reflex was assessed when the dorsal surface of the hind paws contacted the table edge, with the position of the hind limbs slightly below that of the forelimbs. The investigator recorded whether the positioning of the hind paws on the table surface was reflexive or nonreflexive. Righting reflex was evaluated by placing the mice on their backs onto the flat surface. The researcher recorded the rapid return of mice to the natural upright position. Grasping reflex was evaluated with the animals placed onto a wire grid. The investigator recorded the rear paws' wire-on-contact grabbing. All trials were performed five times, with a 10-min interval. Each reflex score was calculated according to the number of normal reflexes observed.

### Alternative RNA splicing analysis by rMATS

**Fig. 1:**
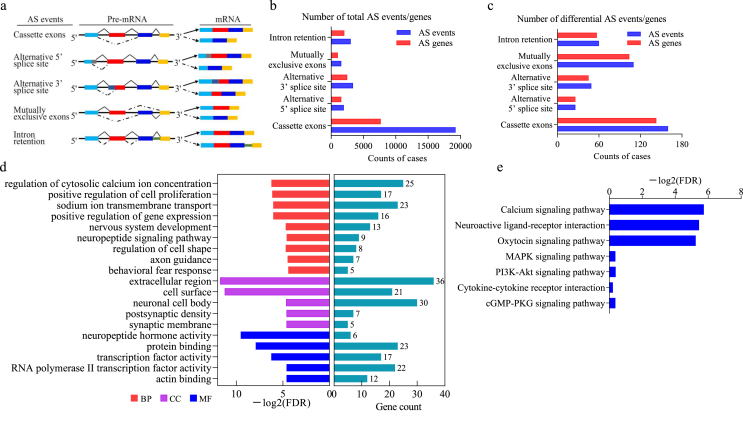
Identification of alternative RNA splicing events and genes in DRGs after SNL. (a) Schematic depicting the five alternative RNA splicing events identified by rMATS. A black line denotes the introns, and exons are shown as blue, red, dark blue, and yellow rectangular objects. Exon skipping is the result of exon exclusion, alternative 5′ splice sites involve shortening of the 5′ exons due to internal splice sites, and alternative 3′ splice sites involve shortening of the 3′ exons due to internal splice sites. Mutually exclusive exons occur when one exon is included while the other has been excised. Intron retention occurs when an intron is retained and not excised. (b) The numbers of total alternative RNA splicing events and corresponding genes between the SNL and sham groups. (c) The numbers of differential AS events and related genes between the SNL and sham groups. (d) Enrichment analysis of the biological process of differential AS compared with the sham group. The biological process (BP), cellular component (CC), and molecular function (MF) categories with FDR＜0.05 are listed. The horizontal axis represents the −log2 (FDR) of the significant GO terms. (e) Enrichment analysis of KEGG pathways of differential AS in SNL compared with the sham group. The horizontal axis represents the –log2 (FDR) of the significant pathways.

Using ENSEMBL gene models, alternative differential splicing was investigated with rMATS v4.0.2. rMATS detects AS events in skipped exon (SE), retained intron (RI), mutually exclusive exon (MXE), alternative 5′ splice site (A5SS), and alternative 3′ splice site categories (see schematic in Fig. 1a). Mapped .bam files were used as input for rMATS. For each alternative RNA splicing event, rMATS calculates the percentage of splicing inclusion (PSI) for each sample across biological triplicates, determines the differential PSI (*Δ*PSI) between two conditions, and outputs two types of data, using reads mapped to splice junctions on its own or utilizing reads mapped to splice junctions and the exon body. We determined the differential alternative RNA splicing events with ΔPSI >0.05 and false discovery rate (FDR) ​< ​0.1 as the thresholds (Supplementary file 1).

### DRG microinjection

DRG microinjection was carried out as previously described [28]. In brief, inhaling 1.5–2% sevoflurane rendered the mice anesthetized, and a dorsal midline incision was performed in the lower lumber back location. Utilizing tiny rongeurs, the left L_4_ and/or L_3_ and L_4_ articular processes were exposed and excised. The viral (1 ​μL, 2 ​× ​0^14^ TU/mL) solution was injected into the unilateral L_4_ and/or L_3_ and L_4_ DRG using a Hamilton syringe linked to a glass micropipette following total exposure of the DRG. After each DRG injection, the glass micropipette was left in place for 10 ​min before removal. The surgical field was filled with sterile saline, and then the incision was closed using metal wound clamps. Animals with abnormal locomotor activity were excluded.

### Cell culture and transfection

DRG neurons and HEK-293T cells were cultured utilizing previously described methods [28,30]. Briefly, HEK-293T cells were cultivated in Neurobasal™-A Medium (Thermo Fisher Scientific) containing 1 ​% antibiotics and 10 ​% fetal bovine serum (FBS) (Thermo Fisher Scientific). To prepare the primary DRG neuron culture, 4-week-old mice were euthanized with 5 ​% sevoflurane, and bilateral DRGs were collected in cold neurobasal medium (Thermo Fisher Scientific) containing 10 ​% FBS and 1 ​% antibiotics. The tissues were incubated with Hanks' balanced salt solution (HBSS) (Thermo Fisher Scientific), including 1 ​mg/mL collagenase type I and 5 ​mg/mL dispase for 2 ​h at 37 ​°C, followed by 10 ​min of digestion using 0.25 ​% trypsin (Sigma) at 37 ​°C and then using 0.25 ​% trypsin inhibitor (Sigma). Dissociated neurons were resuspended in neurobasal-defined medium containing 2 ​% B27 supplement following trituration and centrifugation (Invitrogen). These were then transferred to a 6-well plate containing 50 ​μg/ml poly-D-lysine (Sigma). The neurons were subsequently grown at 37 ​°C and 5 ​% CO_2_. After 24 ​h, 2 ​μl of AAV5 (titer ≥1 ​× ​10^13^ TU/ml) was added to each 2 ​ml well. Three days later, neurons in the culture were harvested for Western blot analysis.

### Reverse transcription quantitative RCR

Total RNA extraction and quantitative real-time RT‒PCR assays were performed as described previously [28,32]. Briefly, sufficient RNA was obtained by pooling together four ipsilateral L_4_ DRG from four SNL mice or four ipsilateral L_3/4_ DRG from two CCI animals. The miRNeasy kit with on-column digestion of genomic DNA (Sigma‒Aldrich LLC) was used to extract total RNA according to the manufacturer's instructions and then reverse-transcribed using ThermoScript reverse transcriptase (Thermo Fisher Scientific) with oligo (dT) primers. The template (1 ​μl) was amplified with a real-time PCR system. Each sample was run three times in a 20 ​μl reaction volume containing 250 ​nM forward and reverse primers, 10 ​μl of SsoAdvanced Universal SYBR Green Supermix (Bio-Rad), and 20 ​ng of cDNA. The PCR amplification included 30 ​s at 95 ​°C, 30 ​s at 60 ​°C, 30 ​s at 72 ​°C, and 5 ​min at 72 ​°C for 38 cycles. Ratios of ipsilateral-side mRNA levels to contralateral-side mRNA levels were computed using the ΔCt method (2^－ΔΔCt^) following normalization against the corresponding *tuba1α*, which was demonstrated to be steady in injured DRGs after peripheral nerve injury. All the primers are presented in Table 1.

**Table 1:** Primer sequences.

Genes	Primer name	Sequences (5′ to 3′)
***RT qPCR***		
*Rbfox1*	F	GGTATGCCGCCAAATCTAAA
	R	GCTGAGGTCCAAAATTTCCA
*Rbm28*	F	GTGCATCTCCATCCATGTTG
	R	GTGGGTTCCAGGTCTACGAA
*Hur*	F	AACACGCTGAACGGCTTGAGGCT
	R	CCGCCCAAACCGAGAGA ACAT
*Brunol5*	F	CTTCTATCCCATCCTGTGAAGC
	R	GGGCATGAAATTAGAAGACAGG
*Pabpc3*	F	GAGAATGAACCCCAGCACCC
	R	ACATACGCGTAGTTGGAGGA
*Srp20*	F	ATGCATCGTGATTCCTGTC
	R	ATAGTGTTCTTCCATCTAGC
*L-Nrcam*	F	GTTTCCCAAGGCCTAAATGG
	R	CACCATAAAACTCAGTGTCACT
*S-Nrcam*	F	GTTTCCCAAGGCCTAAATGG
	R	ACTAGATTTAGCTGAAATCACC
*T- Nrcam*	F	GTTTCCCAAGGCCTAAATGG
	R	AATAGGCTGCTTCTGCTGGA
*Tuba1α*	F	GTGCATCTCCATCCATGTTG
	R	GTGGGTTCCAGGTCTACGAA
***Cloning***		
*Rbfox1*	F	CTCCTCCACCAGACCTCTTG
*Rbfox1*	R	TTCCCACCAATCTGAGCCAT
*Rbfox1*	F	TATGGATCCGCCACCATGGTGTTTTCGATGT
*Rbfox1*	R	GCGCTCGAGCTAGACTGTTTCCTTGAACA

RT: Reverse-transcription; F: Forward; R: Reverse.

**Table 2:** Locomotor function.

Treatment groups	Locomotor function test		
	Placing	Grasping	Righting
AAV5-*Gfp* ​+ ​Sham (male)	5 (0)	5 (0)	5 (0)
AAV5-*Gfp* ​+ ​SNL (male)	5 (0)	5 (0)	5 (0)
AAV5- *Rbfox1* ​+ ​Sham (male)	5 (0)	5 (0)	5 (0)
AAV5- *Rbfox1* ​+ ​SNL (male)	5 (0)	5 (0)	5 (0)
AAV5-*Gfp* ​+ ​Sham (female)	5 (0)	5 (0)	5 (0)
AAV5-*Gfp* ​+ ​SNL (female)	5 (0)	5 (0)	5 (0)
AAV5- *Rbfox1* ​+ ​Sham (female)	5 (0)	5 (0)	5 (0)
AAV5- *Rbfox1* ​+ ​SNL (female)	5 (0)	5 (0)	5 (0)
AAV5-scrambled shRNA (male)	5 (0)	5 (0)	5 (0)
AAV5- *Rbfox1* shRNA (male)	5 (0)	5 (0)	5 (0)
AAV5- scrambled shRNA (female)	5 (0)	5 (0)	5 (0)
AAV5-*Rbfox1* shRNA (female)	5 (0)	5 (0)	5 (0)
SNL ​+ ​AAV5-*Gfp* (male)	5 (0)	5 (0)	5 (0)
SNL ​+ ​AAV5- *Rbfox1* (male)	5 (0)	5 (0)	5 (0)
Sham ​+ ​SON	5 (0)	5 (0)	5 (0)
Sham ​+ ​AON	5 (0)	5 (0)	5 (0)
SNL ​+ ​SON	5 (0)	5 (0)	5 (0)
SNL ​+ ​AON	5 (0)	5 (0)	5 (0)

*n* ​= ​10–12 mice per group; 5 trials; Mean (sem).

Abbreviations: SNL, spinal nerve ligation; SON, scrambled oligonucleotide; AON, *Nrcam* antisense oligonucleotide.

### Western blot analysis

We adopted our previously described procedure for protein extraction and Western blotting [28,30]. To obtain adequate protein, unilateral L_4_ DRGs from four mice or unilateral L_3_ and L_4_ DRGs from two mice were pooled together. The tissues or the cultured cells were homogenized and ultrasonicated in lysis buffer (10 ​mM Tris, 1 ​mM phenylmethylsulfonyl fluoride, 250 ​mM sucrose, 5 ​mM MgCl_2_, 5 ​mM EGTA, 1 ​mM EDTA, 1 ​mM DTT, 40 ​μM leupeptin) containing EDTA-free protease inhibitor cocktail (ROCHE/04693159001) and phosphatase inhibitor cocktail (Abcam/ab201114) for 10 ​min on ice. Approximately 10 ​% of the homogenate (in volume) was used for total protein. The remaining sample was centrifuged at 4 ​°C for 15 ​min at 1000 ​g. After the protein concentration was determined by BCA assay, the samples were heated at 99 ​°C for 5 ​min and loaded onto a 4–20 ​% stacking/7.5 ​% separating SDS-polyacrylamide gel. The proteins were electrophoretically transferred onto a polyvinylidene difluoride membrane. The membrane was blocked with 5 ​% nonfat milk in Tris-buffered saline containing 0.1 ​% Tween-20 for 1 ​h at room temperature; the membrane was incubated with the following primary antibodies overnight at 4 ​°C: rabbit anti-Rbfox1 (1:1000; Abcam), rabbit anti-Rbfox2 (1:1000; Abcam), rabbit anti-GAPDH (1:2000; Abcam), rabbit anti-total ERK1/2 (1:1000; Abcam), rabbit anti-phosphorylated ERK1/2 (p-ERK1/2; 1:1000; Abcam), and mouse anti-GFAP (1:1000; Abcam). Horseradish peroxidase-linked goat-anti-mouse (1:2000, Cell Signaling Technology) or goat-anti-rabbit (1:2,500, ThermoFisher Scientific) secondary antibodies were used to identify the proteins. The proteins were exposed using the ChemiDoc XRS imaging system with Image Lab software (Bio-Rad) and observed using equal parts of Clarity western peroxide agent and Clarity western luminol/enhancer reagent (Bio-Rad). Using densitometry and Image Lab software (Bio-Rad), Western blot intensity was determined. The protein bands were normalized to GAPDH.

### Immunohistochemistry

Immunohistochemistry was performed as reported previously [30]. Briefly, the mice were transcardially perfused with 30 ​ml of 0.01 ​M phosphate-buffered saline (PBS) and then with 50 ​ml of 4 ​% paraformaldehyde in 0.1 ​M phosphate buffer (pH 7.4) after deep sevoflurane anesthesia. The DRGs were collected, postfixed for 6 ​h in fixative solution, and then cryoprotected overnight at 4 ​°C in 30 ​% sucrose in 0.1 ​M phosphate buffer. On a cryostat, DRGs were sectioned to a thickness of 20 ​μm. The sections were blocked for 1 ​h at room temperature with 5 ​% goat serum and 0.5 ​% Triton X-100 in PBS before being incubated overnight at 4 ​°C with anti-Rbfox1 (1:250, Abcam), anti-NeuN (1:250, Cell Signaling Technology), anti-glutamine synthetase (1:500, Abcam), anti-NF200 (1:250, Abcam), anti-CGRP (1:200, Abcam), and biotinylated IB4 (1:250, Sigma). On the second day, the sections were incubated with species-appropriate secondary antibody Alexa Fluor® 568 (1:200, Abcam) or Alexa Fluor® 488 (1:250, Abcam) at room temperature for 2 ​h. The images were captured using a DMI 4000 fluorescence microscope (Leica) with a DFC365 FX camera (Leica) and then processed with ImageJ software.

### Plasmid construction and virus production

To construct the plasmid that is accountable for the expression of the Rbfox1 protein, the full-length sequences of the *Rbfox1* mRNA extracted from mouse DRGs were reverse-transcribed and amplified using PCR and primers (Table 1). After Xhol and Kpnl digestion of the accomplished segment, the segment was inserted into the pro-viral plasmid (pAAV5-MCS). DNA sequencing was utilized to verify the recombinant clones. With the assistance of the AAV-DJ Helper Free Packaging System, AAV-DJ viral particles were manufactured (GENECHEM). The *Rbfox1* shRNA duplex that correlates to nucleotides 13–36 of the mouse Rbfox1 mRNA was constructed (the accession number for this sequence in GenBank is NM 021477). After being produced, the oligonucleotides that contained the shRNA sequences were permitted to anneal. The control consisted of a mismatched shRNA termed scrambled shRNA, which had a muddled-up sequence and no homology to a mouse gene. By utilizing the BamH1/XbaI restriction sites, the fragments were successfully covalently attached to the pro-viral plasmid (pAAV5-U6-shRNA). Using the AAV Pro Purification Kit (All Serotypes), the AAV particles were purified (Takara Biomedical Technology).

### RNA immunoprecipitation

RNA immunoprecipitation (RIP) experiments were performed using the Magna RIP kit (Millipore) in accordance with the manufacturer's protocol. The DRGs of mice were washed with ice-cold PBS before being homogenized and lysed in RIP lysis buffer. The supernatants were collected after homogenization and centrifuged at 14,000 ​rpm for 10 ​min at 4 ​°C. Four-fifths of the supernatants was used for immunoprecipitation, while the remaining fraction was utilized as input. A/G magnetic beads were incubated with anti-Rbfox1 antibody (Abcam) or a negative control mouse IgG antibody (Millipore) for 30 ​min at room temperature, followed by incubation with the supernatants overnight at 4 ​°C with gentle rotation and then washing with ice-cold RIP wash buffer. Immunoprecipitants and inputs were resuspended in proteinase K buffer and incubated for 30 ​min at 55 ​°C under rotating conditions to digest the protein. A phenol–chloroform–isopropanol solution was used to isolate coupled RNA, which was then precipitated with ethanol. qRT-PCR was performed utilizing pure RNA samples as templates. Target mRNA recovery was determined as the ratio of mRNA in Rbfox1 immunoprecipitants to mRNA in tissue extracts and was normalized to actin to compensate for nonspecific RNA pull-down.

### Design for *Nrcam* exon 10 antisense oligonucleotide

The design of *Nrcam* antisense oligonucleotide (AON) was based on previous reports. *Nrcam* AON targeted the 5′ and 3′ splice-sites of exon 10, as well as the sequence adjacent to the 3′ splice-site of intron 9 and 10, to prevent their recognition by the spliceosome. The designed *Nrcam* AON sequence is UGUAACUCACCUGAAAUAAACAGAAUAUCAUGAAGAGGGCACAGAAGUAG. The AON was 2′-O-methyl RNA oligonucleotides. The backbone of AON was modified with 5 phosphorothioates on the 5′ and 3′ ends (Table 3). The scrambled oligonucleotide (SON) was designed based on the *Nrcam* AON sequence without modifications of phosphorothioates. The designed sequences were sent to MCE (MedChemExpress, Inc. Shanghai) for production. The injected AON solution was prepared through mixing 20 ​% Glucose, TurboFect transfection reagent (#2717946) and AON by 20:5:1 in advance.

**Table 3:** Sequences of *Nrcam* antisense oligonucleotide (AON) or scramble oligonucleotide (SON).

Name	Sequence
SON	GCAUGUAAUUCCGGUAGCUACUCGAUUAGCCUAACAAUCC
*Nrcam* AON	U∗G∗U∗A∗A∗C∗UCACCUGAAAUAAACAGAAUAUCAUGAAGAGGGCACAGA∗A∗G∗U∗A∗G

### Statistical analysis

The mice were randomly grouped, and sample sizes were determined based on our prior experience and knowledge with this design. One-way or two-way analysis of variance (ANOVA) or two-tailed independent-sample Student's *t* tests were employed to analyze the data. When ANOVA indicated a significant difference, post hoc Tukey pairwise comparisons of means were performed (GraphPad Prism 8). All data are presented as the mean ​± ​standard error of the mean (sem). The threshold for significance was defined at *P* ​< ​0.05.

## Results

### Identification of alternative RNA splicing events via RNA-Seq in DRGs during neuropathic pain

Alternative RNA splicing is a crucial mechanism in the regulation of gene expression. Accumulating evidence indicates that alternative RNA splicing markedly enhances the functional diversity of the genome. Deviations or alterations in this process yield pathological states [6]. To explore key alternative RNA splicing events related to neuropathic pain after peripheral nerve injury in DRG, we first conducted SNL, a preclinical animal model that simulates peripheral nerve injury. Total RNA was isolated from DRG 7 days after surgery in SNL and sham mice for RNA sequencing (RNA-Seq). The base composition, base sequence quality, and sequence alignment statistics were shown in our earlier work [33]. For genes with multiple transcript isoforms, we examined absolute isoform expression employing rrMATS, an efficient statistical method for robustly and flexibly detecting different alternative RNA splicing events from replicate RNA-Seq data [34]. The splicing events for the five most prevalent types of AS patterns were cataloged: skipped exon (SE), alternative 5′ splice site (A5SS), alternative 3′ splice site (A3SS), mutually exclusive exons (MXE), and retained intron (RI) (Fig. 1a). We found that the distribution of the five types of AS differed between SNL and sham mouse DRG. Over 29,000 alternative RNA splicing events were identified from >14,000 genes. Moreover, the genes with multiple types of alternative RNA splicing might be counted multiple times (Fig. 1b and Supplementary file 1). The SE type of alternative RNA splicing was the most prevalent of the five categories. MXE was the least frequent overall (Fig. 1b). For each alternative RNA splicing type, the SNL group was compared to the sham group to detect differential splicing events with a corresponding change in the PSI of these events. A total of 405 differential alternative RNA splicing events were identified from 375 genes (Fig. 1c and Supplementary file 1).

To investigate the cellular processes and pathways influenced by the altered alternative RNA splicing in mouse DRG, we utilized all different alternative RNA splicing events in DRG from sham *vs.* SNL mice comparisons to perform gene ontology (GO) and pathway analysis via DAVID bioinformatics analysis (DAVID 6.8) [35]. GO analysis revealed several biological processes associated with nociceptive information processing and neural activity, including regulation of cytosolic calcium ion concentration, sodium ion transmembrane transport, neuropeptide signaling pathway, and beneficial regulation of gene expression, with FDR＜0.05 (Fig. 1d, Supplementary file 2). KEGG pathway analysis showed that calcium signaling pathway, oxytocin signaling pathway, and neuroactive ligand‒receptor interaction were statistically significant with a false discovery rate (FDR)＜0.05 (Fig. 1e). Overall, the enrichment of all these biological processes and pathways suggested that alternative RNA splicing plays a major functional role in peripheral nerve injury-induced neuropathic pain in the DRG of mice.

### Rbfox1, a crucial alternative splicing regulator in DRGs, mediates many splicing events during peripheral nerve injury-induced neuropathic pain

**Fig. 2:**
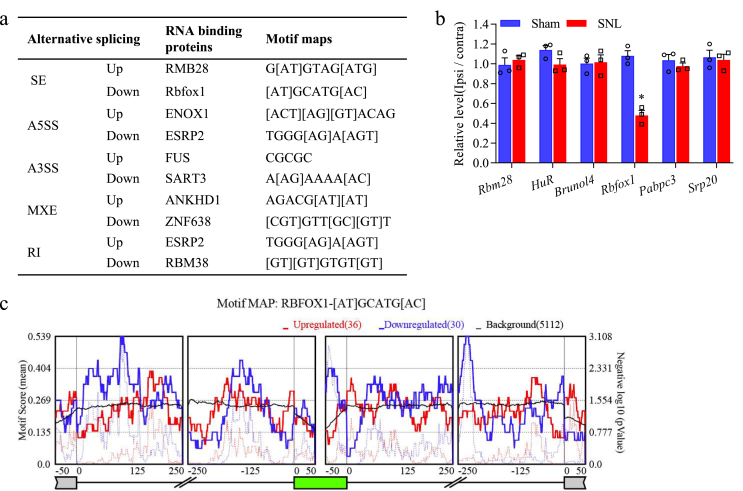
Prediction of splicing regulator RNA binding proteins (RBPs) for each AS event by rMAPS2 in the DRGs of mice with SNL vs. sham surgery. (a) Summary of the RBPs motif maps with the most significant enrichment for each up- and downregulated AS event. (b) Relative mRNA levels of top three enriched RBPs for up- and down-regulated SE events in the ipsilateral L_4_ DRGs of mice after SNL or sham surgery (n ​= ​3 per group). (c) RNA motif maps showing enrichment of Rbfox1 near exons that are alternatively spliced. Maps for Rbfox1 motif enrichment for SE events from the rMATS analysis of SNL mice DRGs are shown for silenced (red) and enhanced (blue) splicing events. The dotted lines indicate the significance of enrichment versus background. The solid lines indicate the motif score of upregulated and downregulated genes compared to background genes.

Alternative RNA splicing is primarily regulated by RBPs, which often work by directly binding to regulatory pre-mRNA sequence motifs in exons and/or surrounding intronic sequences of alternatively spliced exons, in a cell-type-specific manner, particularly in neurons [14]. To identify the explicit RBPs associated with coordinated regulation of each alternative RNA splicing event and to evaluate their potential biological impact in the DRG of SNL and sham mice, we conducted motif enrichment analysis on all differential alternative RNA splicing events that produced rMAPS2 from rMATS. We identified the most substantially enriched RBPs for each up- and downregulated alternative RNA splicing event (Fig. 2a). Since SE is the most common alternative RNA splicing events in the genome of mammals, we focused on the top three enriched RBPs (RBM28, HuR, BRUNOL5, Rbfox1, PABPC3, SRp20) for up- and down-regulated SE events. The mRNA expression levels of *Rbm28*, *HuR*, *Brunol5*, *Pabpc3*, and *Srp20* were not significantly changed in injured DRG after SNL compared to sham surgery. In contrast, *Rbfox1* mRNA expression decreased dramatically (Fig. 2b). Additionally, our previous RNA-seq data also showed that *Rbfox1* was markedly decreased in injured DRG following SNL [33]. These results suggest that Rbfox1 is a crucial splicing regulator of DRG during peripheral nerve injury-induced neuropathic pain. In addition, substantial enrichment of the Rbfox1 motif was detected in the downstream intron regions when the inclusion of the target exon was promoted. In contrast, it was enriched in the upstream flanking intron regions and target exons when the inclusion of the target exon was suppressed (Fig. 2c). This conclusion is in line with the results of a prior study demonstrating that Rbfox1 binding upstream of an alternative exon inhibits exon inclusion, while binding downstream promotes exon inclusion [24,36]. Rbfox1 is thus a potential pre-RNA splicing regulator in injured DRG after SNL.

### SNL decreased Rbfox1 expression in injured DRG after peripheral nerve injury

**Fig. 3:**
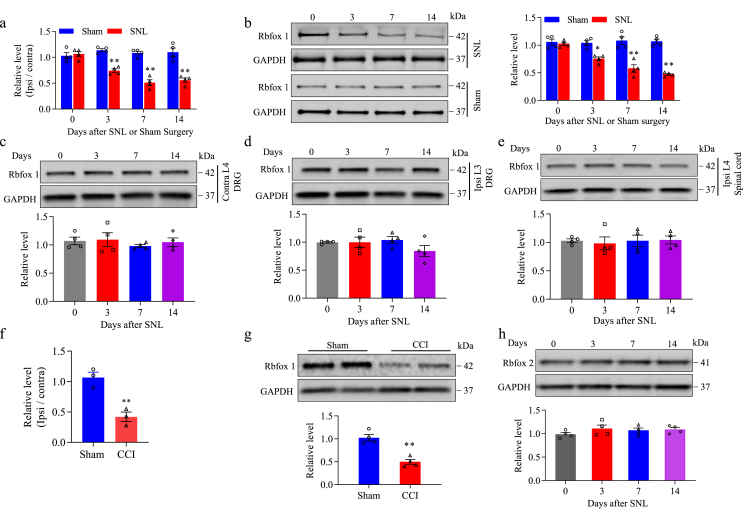
Peripheral nerve injury induced decreased *Rbfox1* mRNA and protein in the injured DRGs. (a) *Rbfox1* mRNA expression in the ipsilateral L_4_ DRG of mice after SNL or sham surgery. n ​= ​12 mice/time point/group. ∗∗*P* ​< ​0.01, according to two-way ANOVA followed by a post hoc Tukey's test. (b) Rbfox1 protein expression in the ipsilateral L_4_ DRG of mice after SNL or sham surgery. Representative Western blots (left panels) and a summary of densitometric analysis (right graphs) are shown. n ​= ​12 mice/time point/group. ∗*P* ​< ​0.05, ∗∗*P* ​< ​0.01, according to two-way ANOVA followed by a post hoc Tukey's test. (c) Rbfox1 protein expression in the contralateral L4 DRG, ipsilateral L3 (intact) DRG, and ipsilateral L4 spinal cord of mice after SNL. Representative Western blots (left panels) and a summary of densitometric analysis (right graphs) are shown. n ​= ​12 mice/time point/group. (d) *Rbfox1* mRNA expression in the ipsilateral L_3/4_ DRGs of mice on day 7 after CCI or sham surgery. n ​= ​6 mice/group. ∗∗*P* ​< ​0.01, according to a two-tailed, independent-sample Student's *t*-test. (e) Rbfox1 protein expression in the ipsilateral L_3/4_ DRGs of mice on day 7 after CCI or sham surgery. n ​= ​6 mice/group. ∗∗*P* ​< ​0.01, according to a two-tailed, independent-sample Student's *t*-test. (f) Rbfox2 protein expression in the ipsilateral L_4_ DRG of mice after SNL or sham surgery. n ​= ​12 mice/time point/group. ∗∗*P* ​< ​0.01, according to two-way ANOVA followed by a post hoc Tukey's test. Representative Western blots (upper panels) and a summary of densitometric analysis (below graphs) are shown.

Peripheral nerve injury leading to neuropathic pain is a chronic progressive process. To further confirm that Rbfox1 expression was altered in injured DRG after unilateral SNL, we examined the mRNA and protein expression of Rbfox1 in SNL mice at different time points. qRT-PCR results indicated that Rbfox1 mRNA in the ipsilateral L_4_ DRG was significantly reduced by 23 ​% on day 3, 51 ​% on day 7, and 46 ​% on day 14 post-SNL surgery compared to the same time points after sham surgery. (Fig. 3a). Western blot results revealed that the levels of Rbfox1 protein were 19 ​% lower on day 3, 45 ​% lower on day 7, and 43 ​% lower on day 14 following SNL compared to those at corresponding time points after sham surgery (Fig. 3b). Neither SNL nor sham surgery altered basal levels of Rbfox1 protein in the contralateral L_4_ DRG (Fig. 3c), the ipsilateral (uninjured) L_3_ DRG (Fig. 3d), or the ipsilateral L_4_ spinal cord dorsal horn (Fig. 3e). Similar results were observed after CCI to the unilateral sciatic nerve, another alternate preclinical animal model of neuropathic pain. On day 7 following CCI, the amount of Rbfox1 mRNA and Rbfox1 protein was lower in the ipsilateral L_3/4_ DRGs than in the corresponding sham animals (Fig. 3f and g), indicating a possible DRG-specific role of Rbfox1 in neuropathic pain. Neither SNL nor sham surgery affected the basal expression of Rbfox2 (another Rbfox family member) protein in injured DRG (Fig. 3h).

**Fig. 4:**
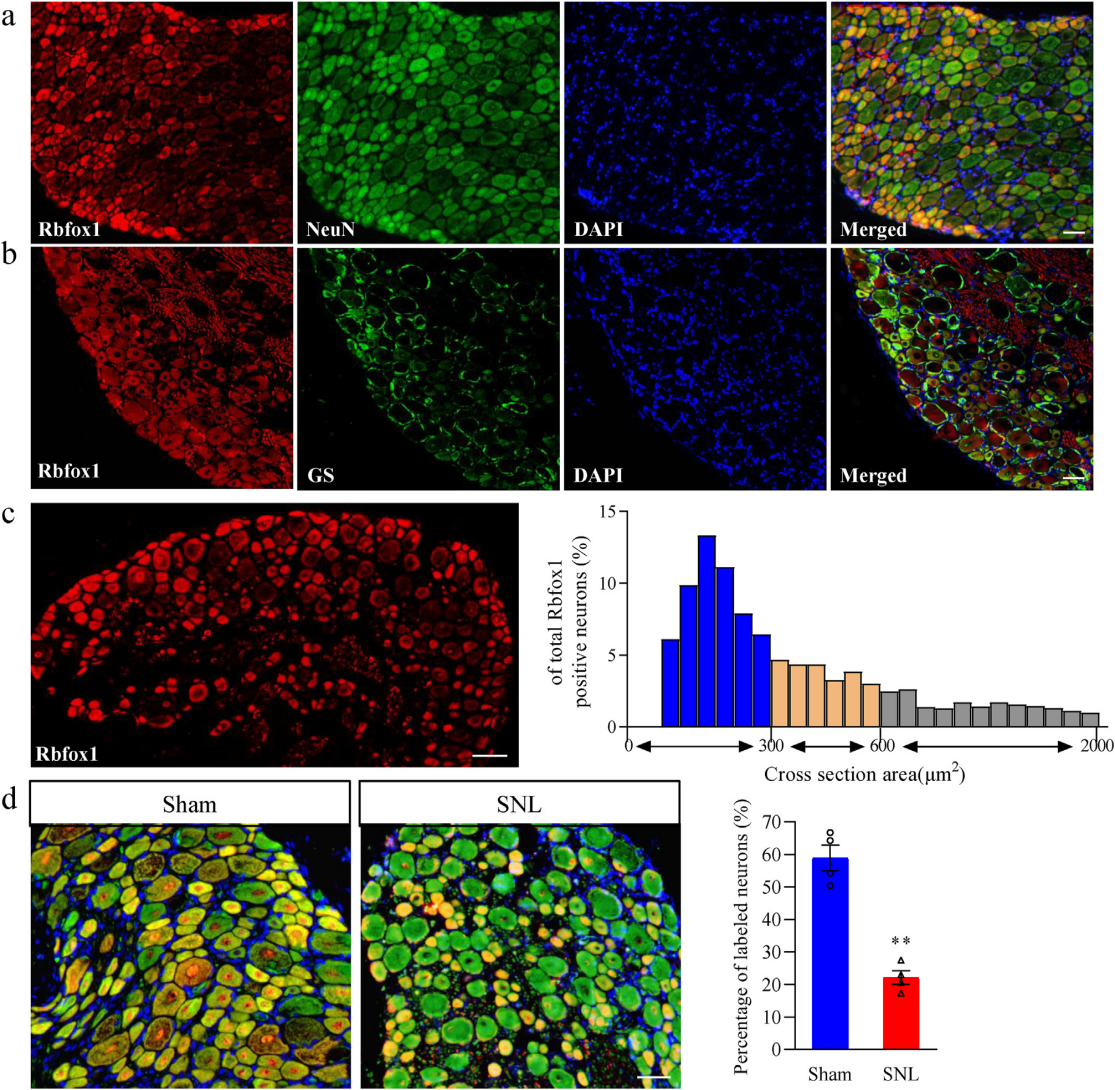
Distribution pattern of Rbfox1 protein in the DRG of naive mice and changes in the number of Rbfox1-positive neurons in injured DRGs after SNL. (a, b) Representative photographs show that Rbfox1 (red color) is coexpressed exclusively with NeuN (a) and undetectable in glutamine synthetase (GS)-labeled cells (b). Cellular nuclei were labeled with 4′,6-diamidino-2-phenylindole (DAPI). n ​= ​3 mice. Scale bars: 200 ​μm for a and b. (c) Histogram displaying the distribution of Rbfox1-positive neuronal somata in DRGs. Small: 58 ​%. Medium: 27 ​%. Large: 17 ​%. (d) Changes in the number of Rbfox1-positive neurons in the ipsilateral L_4_ DRG on day 7 after SNL or sham surgery. n ​= ​3 mice/group. ∗∗*P* ​< ​0.01, according to a two-tailed, independent-sample Student's *t*-test. Scale bar: 100 ​μm.

**Fig. 5:**
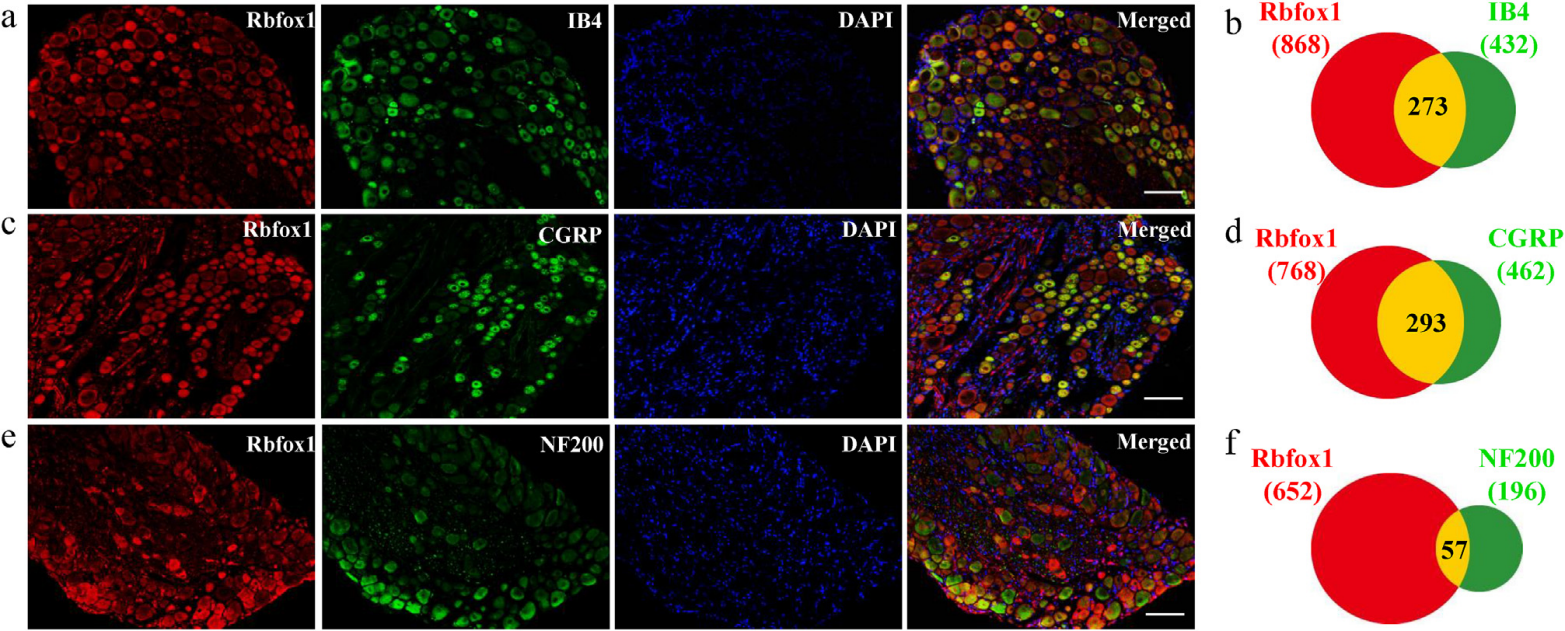
DAPI-stained images of the Rbfox1 protein and several neuronal markers in the DRGs. (a) Rbfox1-positive neurons were labeled by isolectin B4 (IB4), (b) Venn diagram shows the number of neurons double stained by Rbfox1 protein and IB4. (c) Rbfox1-positive neurons were labeled by calcitonin gene-related peptide (CGRP), (d) Venn diagram shows the number of neurons double stained by Rbfox1 protein and CGRP. (e) Rbfox1-positive neurons were labeled by neuroﬁlament-200 (NF200) in naive DRG (n ​= ​3 mice). Scale bars: 200 ​μm. (f) Venn diagram shows the number of neurons double stained by Rbfox1 protein and NF200. n ​= ​3 mice.

In addition, we determined the distribution pattern of Rbfox1 and the change in the number of Rbfox1-positive cells in DRG after SNL. Throughout every DRG cell, Rbfox1 was found to exclusively colocalize with NeuN (a specific neuronal marker) (Fig. 4a). It was undetected in cells labeled with glutamate synthetase (GS, a marker for satellite glial cells) (Fig. 4b). Approximately 75.3 ​% of NeuN-labeled DRG neurons were Rbfox1-positive. In addition, a cross-sectional area assessment of DRG neuronal somata revealed that approximately 51.4 ​% of Rbfox1-labeled neurons were small (area <300 ​μm^2^), 30.5 ​% were medium (300 ​≤ ​area ≤600 ​μm^2^), and 18.1 ​% were large (area >600 ​μm^2^) (Fig. 4c). In accordance with the Western blot results above (Fig. 3b), the number of Rbfox1-labeled neurons in the ipsilateral L_4_ DRG was reduced by 39.4 ​% on day 7 after SNL compared to the corresponding sham group (Fig. 4d). Further analysis revealed that approximately 31.4 ​% of Rbfox1-labeled neurons were positive for isolectin B4 (IB4, a marker for small nonpeptidergic neurons) (Fig. 5a and b), 38.2 ​% were positive for calcitonin gene-related peptide (CGRP, a marker for small peptidergic neurons) (Fig. 5c and d) and 11.8 ​% were positive for neurofilament 200 (NF200, a marker for medium/large cells and myelinated Aβ fibers, Fig. 5e and f). Given that DRG neurons can be categorized into peptidergic and non-peptidergic types, we undertook a detailed examination of the SNL-induced reduction in Rbfox1 expression within these distinct neuronal subtypes. Following 7 days post-SNL, only 22.4 ​% of the neurons labeled with Rbfox1 were identified as non-peptidergic, while 18.2 ​% were characterized as peptidergic (Supplementary Fig. 1). These results suggest a pronounced decline in Rbfox1 expression in both peptidergic and non-peptidergic neuronal subsets after nerve injury.

Taken together, these results indicate that injured DRG-specific and continuing time-dependent downregulation of Rbfox1 may be crucial in the development and maintenance of neuropathic pain behavior.

### Rescuing diminished DRG Rbfox1 attenuates the development of SNL-induced pain hypersensitivity

**Fig. 6:**
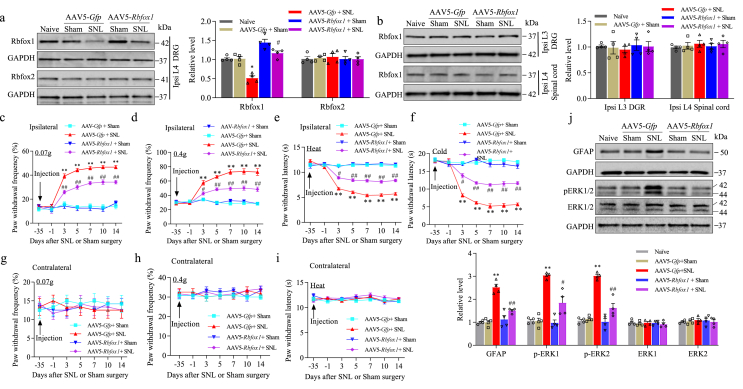
Rescuing diminished DRG Rbfox1 inhibits the development of pain hypersensitivity and spinal cord dorsal horn central sensitization in male mice induced by SNL. (a, b) Rbfox1 and Rbfox2 protein expression in the ipsilateral L_4_ DRG (a) and the level of Rbfox1 protein in the ipsilateral L_3_ DRG and ipsilateral L_4_ spinal cord (b) on day 14 following SNL or sham surgery in male mice microinjected with AAV5-*Rbfox1* or AAV5-*Gfp*. n ​= ​12 mice/group. ∗*P* ​< ​0.05 *vs*. sham male mice microinjected with AAV5-*Gfp* at the corresponding time point. #*P* ​< ​0.05 *vs*. SNL male mice microinjected with AAV5-*Gfp* at the corresponding time point. One-way ANOVA followed by a post hoc Tukey's test. (c–i) Paw withdrawal frequency in response to low (0.07 ​g; c, g) and median (0.4 ​g; d, h) force von Frey filament stimuli and paw withdrawal latency in response to heat (e, i) and cold (f) stimuli on the ipsilateral side (c–f) and contralateral side (g–i) of male mice with microinjection of AAV5-*Rbfox1* or AAV5-*Gfp* into the ipsilateral L4 DRG at different days following SNL or sham surgery. n ​= ​12 mice/group. ∗∗*P* ​< ​0.01 *vs*. sham male mice microinjected with AAV5-*Gfp* at the corresponding time point. #*P* ​< ​0.05, ##*P* ​< ​0.01 *vs*. SNL male mice microinjected with AAV5-*Gfp* at the corresponding time point. Two-way repeated measures ANOVA followed by a post hoc Tukey's test. (I) The levels of phosphorylated ERK1/2 (p-ERK1/2), total ERK1/2, and GFAP in the ipsilateral L_4_ spinal cord dorsal horn of male mice on day 14 after SNL or sham surgery. n ​= ​3 mice/group. ∗∗*P* ​< ​0.01, according to two-way ANOVA followed by Tukey's post hoc test.

Does the reduced Rbfox1 in injured DRG contribute to the pain hypersensitivity caused by nerve trauma in SNL mice? To address this question, we examined the effect of rescuing Rbfox1 on SNL-induced pain hypersensitivity. Considering that AAV5-mediated gene transfer requires 2–4 weeks for activation [28], AAV5 expressing full-length *Rbfox1* (AAV5-*Rbfox1*) was microinjected into unilateral L_4_ DRGs 35 days prior to SNL or sham surgery. As a control, AAV5 expressing green fluorescent protein (AAV5-*Gfp*) was used. Consistent with the above observation, the expression of Rbfox1 protein was reduced by 47.5 ​% in the ipsilateral L_4_ DRG of the AAV5-*Gfp*-microinjected SNL male mice on day 14 following SNL compared to that in the AAV5-*Gfp*-microinjected sham male mice (Fig. 6a). This reduction was significantly mitigated in male SNL mice microinjected with AAV5-*Rbfox1* (Fig. 6a). Neither virus affected the basal level of Rbfox2 in the ipsilateral L_4_ DRG of SNL or sham mice (Fig. 6a), nor did they affect the basal level of Rbfox1 in the ipsilateral L_3_ DRG and ipsilateral L_4_ spinal cord dorsal horn of SNL or sham male mice (Fig. 6b). In line with previous reports [28], SNL induced mechanical allodynia as demonstrated by increases in paw withdrawal frequencies in response to mechanical stimuli (0.07 ​g and 0.4 ​g von Frey filaments), heat and cold hyperalgesia as evidenced by reductions in paw withdrawal latencies in response to heat and cold stimuli, respectively, from days 3–14 after SNL surgery on the ipsilateral (but not contralateral) side of the AAV5-*Gfp*-microinjected SNL male mice (Fig. 6c–i). As anticipated, microinjection of AAV5-*Rbfox1* into the ipsilateral DRG dramatically diminished SNL-induced mechanical allodynia, heat, and cold hyperalgesia (Fig. 6c–i). Neither AAV5-*Rbfox1* nor AAV5-*Gfp* affected the baseline paw withdrawal responses to mechanical, heat, and cold stimulation on the contralateral side of SNL male mice and both the ipsilateral and contralateral sides of sham male mice (Fig. 6c–i). Furthermore, these SNL or sham male mice receiving AAV5-*Rbfox1* or AAV5-*Gfp* pre-microinjection into the DRG exhibited normal locomotor activity (Table 2).

In the ipsilateral lumbar enlargement segment, peripheral noxious stimuli or nerve injury triggered elevation of phosphorylation of ERK 1/2 (pERK1/2, a marker of neuronal hyperactivation) and glial fibrillary acidic protein (GFAP, a marker of astrocyte hyperactivation). To further validate the abovementioned behavioral observations, we also explored whether SNL-induced pERK1/2 and GFAP in the ipsilateral L_4_ spinal cord dorsal horn could be inhibited by microinjection of AAV5-*Rbfox1* into the DRG of male mice. In accordance with the findings of earlier studies [30,37], SNL significantly elevated the levels of pERK1/2 (but not total ERK1/2) and GFAP in comparison to those in the sham group (Fig. 6j). Microinjection of AAV5-*Rbfox1* reduced SNL-induced elevation of pERK1/2 and GFAP expression in the spinal cord dorsal horn of the SNL male mice (Fig. 6j). AAV5-*Rbfox1* microinjection into the DRG of sham male mice did not affect baseline levels of p-ERK1/2 and GFAP in the ipsilateral L_4_ of the spinal cord dorsal horn (Fig. 6j).

Similar results were observed in SNL/sham female mice microinjected with AAV5-*Rbfox1* or AAV5-*Gfp* into the DRG (Supplementary Figure 2a-h and Table 2). Collectively, the above findings clearly demonstrate that the reduced Rbfox1 in injured DRGs of both male and female mice is essential for neuropathic pain development.

### Rescuing diminished DRG Rbfox1 attenuates the maintenance of SNL-induced pain hypersensitivity

**Fig. 7:**
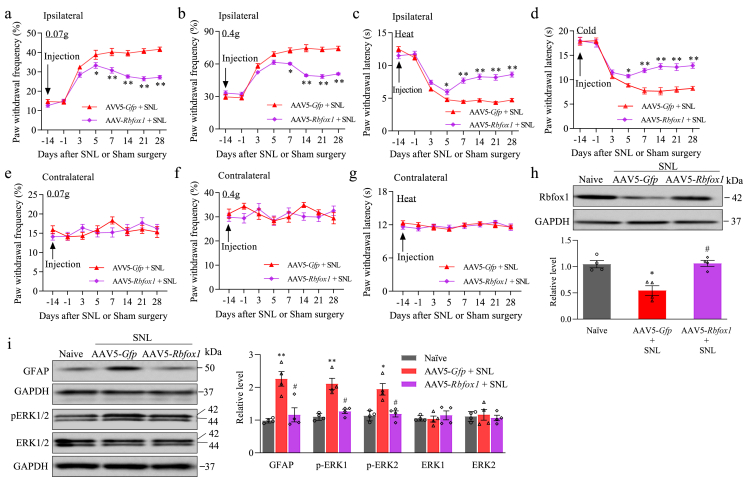
Rescuing the reduction in DRG Rbfox1 caused by SNL mitigated the maintenance of SNL-induced pain hypersensitivity and spinal cord dorsal horn central sensitization in male mice. Male mice were subjected to SNL 14 days following DRG microinjection of AAV5-*Rbfox1* or AAV5-*Gfp*. (a–g) Paw withdrawal frequency in response to low (0.07 ​g; a, e) and median (0.4 ​g; b, f) force von Frey filament stimuli and paw withdrawal latency in response to heat (c, g) and cold (d) stimuli on both the ipsilateral (a–d) and contralateral (e–g) sides of male mice with microinjection of AAV5-*Rbfox1* or AAV5-*Gfp* into the ipsilateral L_4_ DRG at different days after SNL. N ​= ​12 mice/group. ∗*P* ​< ​0.05, ∗∗*P* ​< ​0.01 vs. SNL male mice microinjected with AAV5-*Gfp* at the corresponding time point on the ipsilateral side. Two-way repeated measures ANOVA followed by a post hoc Tukey test. (h) Rbfox1 protein in the ipsilateral L_4_ DRG of naive and AAV5-*Rbfox1-*or AAV5-*Gfp*-microinjected male mice on day 28 following SNL or sham surgery. n ​= ​12 mice/group. ∗*P* ​< ​0.05 vs. naive male mice. #*P* ​< ​0.05 vs. SNL male mice microinjected with AAV5-*Gfp*. One-way ANOVA followed by Tukey's post hoc test. (I) Total ERK1/2, p-ERK1/2 and GFAP levels in the ipsilateral L_4_ spinal cord dorsal horn on day 28 following SNL or sham surgery in male mice with DRG microinjection of AAV5-*Rbfox1* or AAV5-*Gfp*. n ​= ​3 mice/group. ∗*P* ​< ​0.05, ∗∗*P* ​< ​0.01 vs. naive male mice. #*P* ​< ​0.05 vs. SNL male mice microinjected with AAV5-*Gfp*. One-way ANOVA followed by Tukey's post hoc test.

**Fig. 8:**
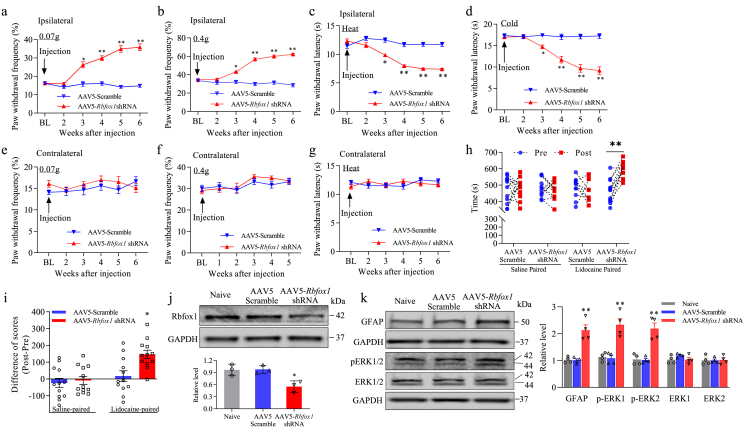
DRG Rbfox1 knockdown produced an enhanced pain response and spinal cord dorsal horn central sensitization in naive male mice. (a) The level of Rbfox1 in the ipsilateral L_3/4_ DRGs 7 weeks after microinjection of AAV5-*Rbfox1* shRNA or AAV5-Scramble shRNA. n ​= ​10 mice/group. ∗*P* ​< ​0.05 vs. male mice microinjected with AAV5-Scramble shRNA. One-way ANOVA followed by Tukey's post hoc test. (b–h) Effect of microinjection of AAV5-*Rbfox1* shRNA or AAV5-Scramble shRNA into the unilateral L_3_/_4_ DRGs on paw withdrawal frequencies in response to low (0.07 ​g; b, f) and median (0.4 ​g; c, g) force von Frey filament stimuli and paw withdrawal latencies in response to heat (d, h) and cold stimuli (e) on both the ipsilateral (b–e) and contralateral (f–h) sides at different weeks after AAV5 microinjection. n ​= ​10 mice/group. ∗*P* ​< ​0.01, ∗∗*P* ​< ​0.01 *vs*. naive mice microinjected with AAV5-Scramble shRNA at the corresponding time points. Two-way repeated measures ANOVA followed by Tukey's post hoc test. (i, j) Effect of unilateral L_3/4_ DRGs microinjected with AAV5-*Rbfox1* shRNA or AAV5-Scramble shRNA on spontaneous ongoing pain as evaluated by the CPP paradigm. n ​= ​10 mice/group. ∗∗*P* ​< ​0.01, according to two-tailed, independent Student's *t*-test. (k) Effect of unilateral L_3/4_ DRG microinjection with AAV5-*Rbfox1* shRNA or AAV5-Scramble shRNA on spinal cord dorsal horn neuronal and astrocyte hyperactivities evidenced by increases in the abundance of p-ERK1/2 and GFAP, respectively, in the ipsilateral L_3/4_ spinal cord dorsal horn 7 weeks after viral microinjection. n ​= ​10 mice/group. ∗∗*P* ​< ​0.01, one-way ANOVA followed by Tukey's post hoc test.

The effects of decreased expression of Rbfox1 in injured DRG on the maintenance of SNL-induced pain hypersensitivity were also tested. AAV5-*Rbfox1* or AAV5-*Gfp* was microinjected into the ipsilateral L_4_ DRG 14 days before SNL surgery in male mice. Mechanical, heat and cold pain hypersensitivities were tested on days 3, 5, 7, 14, 21, and 28 after SNL on the ipsilateral and contralateral sides in both AAV5-*Rbfox1-*and AAV5-*Gfp*-microinjected male mice (Fig. 7a–g). However, these pain hypersensitivities were considerably blunted on days 7, 14, 21, and 28 following SNL in AAV5-*Rbfox1*-microinjected male mice compared to AAV5-*Gfp*-microinjected male mice (Fig. 7a–d). Predictably, SNL reduced the level of Rbfox1 protein by 47.5 ​% in the ipsilateral L_4_ DRG of AAV5-*Gfp*-microinjected SNL male mice 28 days after SNL (Fig. 8h). However, this reduction was absent in SNL male mice microinjected with AAV5-*Rbfox1* (Fig. 8h). Additionally, SNL-induced elevations in p-ERK1/2 (but not total ERK1/2) and GFAP in the ipsilateral L_4_ spinal cord dorsal horn on day 28 after SNL observed in the AAV5-*Gfp*-microinjected SNL male mice were not observed in the AAV5-*Rbfox1*-microinjected SNL male mice (Fig. 8i). The above results reveal the crucial role of reduced DRG Rbfox1 in the maintenance of SNL-induced pain hypersensitivity.

### Mimicking the peripheral nerve injury-induced decrease in DRG Rbfox1 leads to pain hypersensitivity

To further determine whether the reduced expression of Rbfox1 in DRG was sufficient to trigger neuropathic pain following peripheral nerve injury, we mimicked the peripheral nerve injury-induced reduction in DRG Rbfox1 by microinjection of AAV5-*Rbfox1* shRNA into the ipsilateral L_3_ and L_4_ DRG of naive mice. As a control, AVV5-scrambled shRNA was used. As predicted, DRGs microinjection of AAV5-*Rbfox1* shRNA, but not AVV5-scrambled shRNA, triggered substantial increases in paw withdrawal frequencies in response to 0.07 ​g and 0.4 ​g von Frey filament stimuli and decreases in paw withdrawal latencies in response to heat and cold stimuli from week 3 to at least week 7 post-microinjection on the ipsilateral side (Fig. 8a–d). virus did not change the contralateral basal responses (Fig. 8e–g) or locomotor function (Table 2). In addition, microinjection of AAV5-*Rbfox1* shRNA into the DRG evoked stimulation-independent pain hypersensitivity, as illustrated by the CPP paradigm. Mice obviously preferred the lidocaine-paired chamber (specifically, they spent more time in the lidocaine-paired chamber) on week 7 after AAV5-*Rbfox1* shRNA microinjection into the DRGs (Fig. 8h and i). In addition to evoking pain hypersensitivity, the expression of Rbfox1 protein was decreased by 50.4 ​% in the ipsilateral L_3/4_ DRGs of male mice microinjected with AAV5-*Rbfox1* shRNA compared to those microinjected with AVV5-scrambled shRNA 7 weeks after microinjection (Fig. 8j). As predicted, mice with DRGs microinjection of AVV5-scrambled shRNA did not exhibit a significant preference for the saline- or lidocaine-paired chamber, demonstrating the absence of spontaneous pain (Fig. 8h and i). DRGs microinjection of AAV5-*Rbfox1* shRNA, but not AVV5-scrambled shRNA, also elevated the levels of p-ERK1/2 (but not total ERK1/2) and GFAP in the ipsilateral L_3/4_ spinal cord dorsal horn of male mice 7 weeks after microinjection (Fig. 8k), further validating our behavioral observations described above.

Similar results were observed in naive female mice whose DRGs were microinjected with AAV5-*Rbfox1* shRNA or AVV5-scrambled shRNA (Supplementary Fig. 3a-j and Table 2). Collectively, our results showed that downregulating mouse DRG Rbfox1 is sufficient to both evoke spontaneous and trigger pain hypersensitivities, characteristic symptoms of neuropathic pain often seen in the clinic, even in the absence of peripheral nerve injury.

### Rbfox1 modulates neuropathic pain by regulating alternative *Nrcam* Splicing

**Fig. 9:**
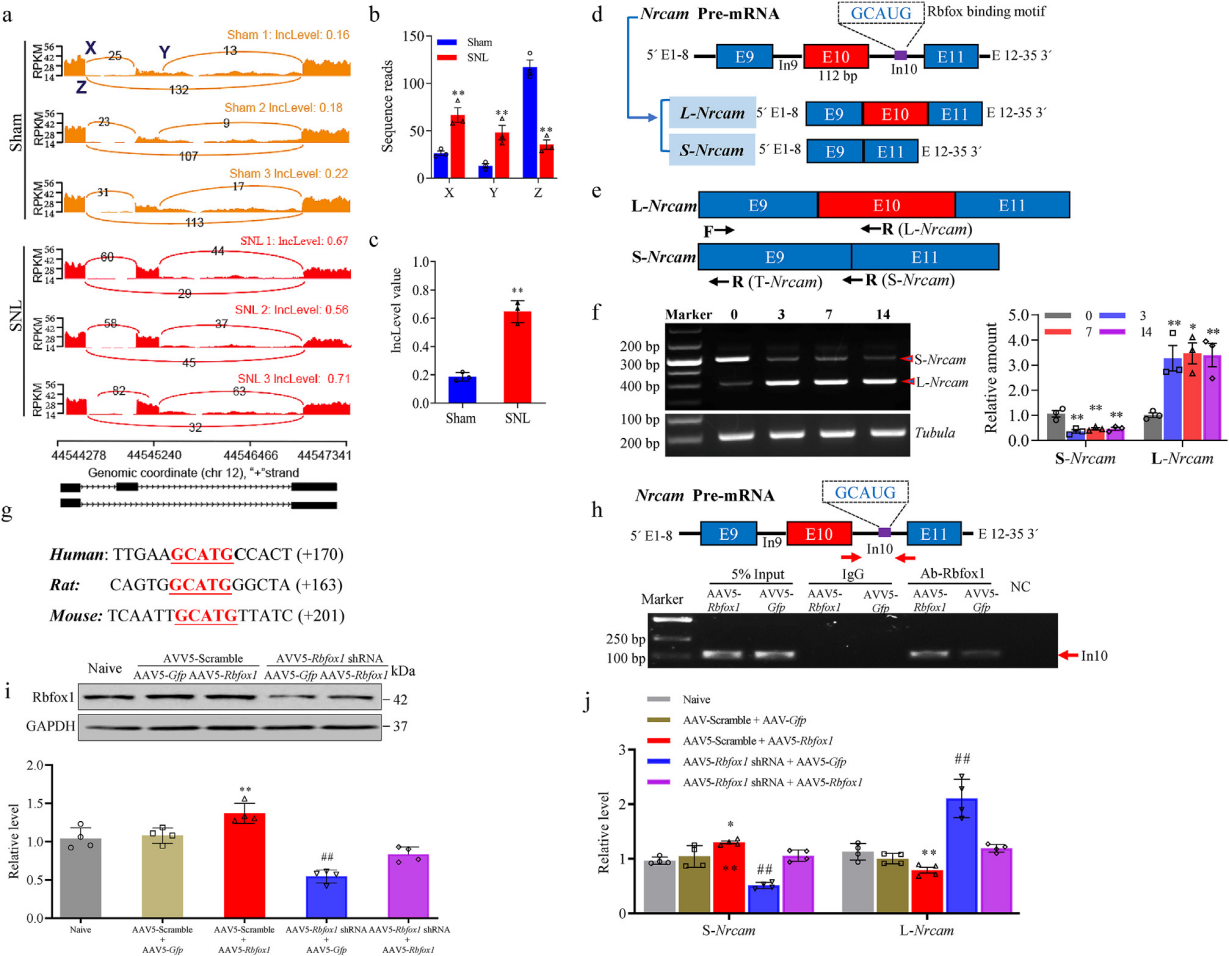
SNL led to an increase in l*Nrcam* mRNA expression in injured DRGs. (a) Exon 10 inclusion was increased by SNL, and the rMATS model revealed exon 10 inclusion reads (X and Y) and exclusion reads (Z) for each sample from the SNL and sham groups. (b) SNL drastically decreased exclusion reads (Z), and enhanced inclusion reads (X and Y) of *Nrcam* exon 10. n ​= ​12 mice/group. ∗∗*P*＜0.01 *vs*. sham group according to Student's *t*-test. (c) The lncLevel value increased significantly in the SNL group compared to sham group. The lncLevel value, which indicates “percentage spliced in” denoting the fraction of mRNAs that represent the inclusion isoform. n ​= ​3 biological replicates (12 mice/group). ∗∗*P* ​< ​0.01 *vs* the sham group according to *t*-test. (d) Schematic of *Nrcam* gene splicing variants at exon 10, *Nrcam* pre-mRNA two splicing variants L-*Nrcam* (inclusion exon 10) and S-*Nrcam* (exclusion exon 10). (e) Schematic graph showing the position of primers used in PCR reactions and qRT-PCR to examine total *Nrcam* mRNA and its variants. (f) Typical images (left panels) demonstrating elevated L-*Nrcam* and diminished S-*Nrcam* transcripts in injured DRGs after SNL by summary of qPCR densitometric analysis (right graphs). There were significant increases in the level of L-*Nrcam* mRNA and corresponding decreases in the amount of S*-Nrcam* mRNA in injured L_4_ DRGs at 3, 7, and 14 days after SNL compared with the sham group. n ​= ​12 mice/group. One-way ANOVA followed by Tukey's post hoc test. ∗*P* ​< ​0.05, ∗∗*P* ​< ​0.01 *vs*. the corresponding control group (0 days). (g) Sequence containing Rbfox1 binding motif in *Nrcam* in human, rat, and mouse. Number represents location from intro 10. (h) Cross-link RNA immunoprecipitation assay of *Nrcam* pre-mRNA sequence. Cultured DRG neurons were infected with AVV5-*Rbfox1* and compared with the AAV5-*Gfp* infected cultured DRG neurons. Rbfox1 binding to regions of *Nrcam* pre-mRNA was detected by semiquantitative RT-PCR as indicated. NC: negative control (H_2_O). Input: total purified *Nrcam* mRNA fragments from the cultured DRG neurons. n ​= ​3 biologic repeats/group. *∗∗P* ​< ​0.01, according to 2-tailed, unpaired Student's *t*-test. (i) Levels of Rbfox1 were assessed in mouse cultured DRG neurons following the indicated transductions. n ​= ​4 biological repeats/treatment. One-way ANOVA followed by Tukey's post hoc test. *∗∗P* ​< ​0.01 vs Naive. ##*P* ​< ​0.01 vs the AAV5 Scramble ​+ ​AAV5-*Gfp* treatment. (j) mRNA expression S-*Nrcam* and L-*Nrcam* were examined in mouse cultured DRG neurons under the specified transductions. n ​= ​4 biological repeats/treatment. One-way ANOVA followed by Tukey's post hoc test.

To further investigate how reduced DRG Rbfox1 contributed to neuropathic pain caused by peripheral nerve injury. We analyzed differentially expressed alternative splicing genes in both SNL and sham mouse DRG. Intriguingly, among these differentially expressed alternative splicing genes, *Nrcam* exon 10, located at Chr12, from 44545379 to 44545435, and includes 57 bases (5′-TGGATGAATTGAATGACACTATAGCTGCTAATTTGAGTGACACTGAGTTTTATGGTG-3′) encoding amino acids from 235 to 253 (VDELNDTIAANLSDTEFYGA) of NrCAM. inclusion variants (L-*Nrcam*) showed significantly increased expression. In contrast, the *Nrcam* exon 10 exclusion variant (S-*Nrcam*) showed decreased expression in injured DRG in the SNL group compared with the sham group (Fig. 9a–c). Our previous studies have identified two *Nrcam* RNA variants in mouse DRG, with SNL induced an increase in expression of the L-*Nrcam* variant in injured DRG [32]. Full-length *Nrcam* cDNA encodes NrCAM (NgCAM-related cell adhesion molecule), an immunoglobulin (Ig) superfamily adhesion molecule that is known to play a role in axon growth and repulsion [38]. We hypothesized that NrCAM might be related to Rbfox1 molecular function, so we searched for the Rbfox1 binding motif in the *Nrcam* mRNA sequence. Notably, we identified a consensus motif sequence (GCAUG) with a predicted Rbfox1 binding motif in the downstream intron of exon 10 of *Nrcam* (Fig. 9d). Quantitative real-time PCR assay with specific primers for L-*Nrcam* and S-*Nrcam* variants, respectively, found that the PCR product intensity of L-*Nrcam* and S-*Nrcam* mRNA exhibited contrasting changes following SNL (Table 1 and Fig. 9e). On days 3, 7, and 14 after SNL, the level of L-*Nrcam* mRNA was significantly upregulated, while the level of S-*Nrcam* mRNA was correspondingly downregulated in injured DRGs (Fig. 9f). Furthermore, motif analysis unveiled a consensus Rbfox1 binding motif (GCAUG) in the downstream intron of exon 10 of human, rat, and mouse *Nrcam* gene (Fig. 9g). Cross-link RNA immunoprecipitation and RT-PCR analysis in cultured DRG neurons, we found that, specific enrichment of Rbfox1 binding to downstream intron of the *Nrcam* gene exon 10 (Fig. 9h). In addition, cultured DRG neurons co-transduced with AAV5-*Rbfox1* shRNA plus AAV5-*Gfp*, but not AAV5-Scramble plus AAV5-*Gfp*, exhibited a significant reduction in Rbfox1 level ((Fig. 9i). Simultaneously, there was a concomitant decrease in the expression of S-*Nrcam* mRNA and an increase in the expression of L-*Nrcam* mRNA compared with the naive group (Fig. 9j). Importantly, these observed changes were not evident in cultured DRG neurons co-transduced with AAV5-*Rbfox1* shRNA plus AAV5-*Rbfox1* (Fig. 9i and j). Conversely, co-transduction of AAV5-*Rbfox1* plus AAV5-Scramble resulted in elevated levels of Rbfox1, leading to an increase in the expression of S-*Nrcam* mRNA and a decrease in the expression of L-*Nrcam* mRNA compared with the naive group in cultured DRG neurons (Fig. 9i and j). In summary, all of this evidence suggests that the *Nrcam* exon 10 is a direct downstream target of Rbfox1-mediated splicing regulation in DRG neurons.

### Nrcam antisense oligonucleotide (AON) attenuates the pain hypersensitivities in neuropathic pain model

**Fig. 10:**
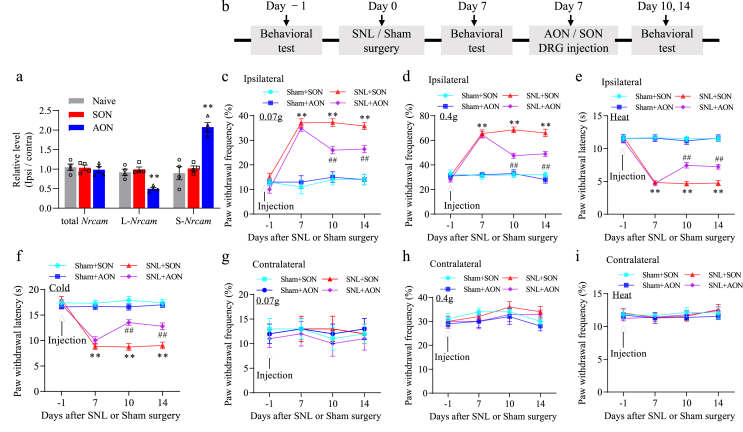
DRG microinjection of *Nrcam* antisense oligonucleotide (AON) relieved SNL-induced neuropathic pain in male mice. (a) Administration with *Nrcam* AON had no effect on total *Nrcam* mRNA expression but decreased the level of L-*Nrcam* mRNA and correspondingly increased the level of S-*Nrcam* mRNA in the cultured DRG neurons. n ​= ​8 mice (4 biological replicates)/group. One way ANOVA followed by Tukey post hoc test. ∗∗*P* ​< ​0.01 vs naive or scramble oligonucleotide (SON) group. (b) The protocols of behavioral tests, surgery, and drug administration. (c-i) *Nrcam* AON reduced the SNL-induced increases in paw withdrawal frequencies to 0.07 ​g (c) and 0.4 ​g ​(d) von Frey filament stimuli and paw withdrawal latencies to thermal (e) or cold (f) stimulation on the ipsilateral side on day 10 and 14 following SNL surgery in male mice, compared with SON treated-SNL male mice. n ​= ​10 male mice/group. Two-way ANOVA (effect *vs* group ​× ​time interaction) followed by post hoc Tukey test., ∗∗*P* ​< ​0.01 *vs* the sham ​+ ​SON. ##*P* ​< ​0.01 *vs* the SNL ​+ ​SON group. The contralateral paw withdrawal frequencies and paw withdrawal latencies were unaffected by AON treatment in male mice (g-i). n ​= ​10 male mice/group. Two-way ANOVA (effect *vs* group ​× ​time interaction) followed by post hoc Tukey test. ∗∗*P* ​< ​0.01 *vs* the sham ​+ ​SON. ##*P* ​< ​0.01 *vs* the SNL ​+ ​SON group.

To regulate exon 10 splicing expression, we designed splice-site 2′-O-methyl modified AON to specifically target the 5′ and 3′ splice-sites of exon 10, as well as the sequence adjacent to the 3′ splice-site of intron 9 and 10 (Table 3). *Nrcam* AON could induce elevated level of exon 10 skipping and expression of S*-Nrcam*. The scrambled oligonucleotide (SON) was used as a control (Table 3). On cultured DRG neurons, the effect of *Nrcam* AON on exon 10 insertion was validated. As shown (Fig. 10a), 100 ​nM *Nrcam* AON had no effect on the total *Nrcam* RNA level, but significantly decreased L-*Nrcam* expression level and correspondingly increased the S*-Nrcam* expression level. Compared to the naive group, *Nrcam* AON reduced the amount of L-*Nrcam* mRNA by 46 % and increased the level of S*-Nrcam* mRNA by 2.2-fold. This expressional shift suggests that *Nrcam* AON inhibits exon 10 insertion and consequently leads to more S*-Nrcam* mRNA variant. As expected, in contrast to the naive group, the SON had no such effect (Fig. 10a). Our results indicate that the effect of alternative splicing on *Nrcam* AON is sequence specific.

Next, we investigated whether *Nrcam* AON could mitigate the SNL-induced neuropathic pain. The AON and SON (5 ​μM, 1 ​μl) were microinjected into the ipsilateral L4 DRG on days 7 when the mechanical allodynia, thermal hyperalgesia, and cold allodynia have been established in the SNL mice (Fig. 10b). The mechanical, thermal, and cold tests were examined at days 10 and 14 after SNL or sham surgery. SNL led to mechanical allodynia, thermal hyperalgesia, and cold allodynia on the ipsilateral (but not contralateral) side in the SNL ​+ ​SON treated group on days 10 and 14 after SNL (Fig. 10c–f). These pain hypersensitivities were ameliorated on the ipsilateral side in the SNL ​+ ​*Nrcam* AON treated group on days 10 and 14 post-SNL (Fig. 10c–j). DRG microinjection of SON had no effect on the SNL-induced mechanical allodynia, thermal hyperalgesia, and cold allodynia on the ipsilateral side during observation period (Fig. 10c–j). As anticipated, neither *Nrcam* AON nor SON affected basal threshold to mechanical, thermal, or cold stimulus on the contralateral side (Fig. 10c–j). To exclude the possibility that the antinociceptive effects of *Nrcam* AON were produced by impaired locomotor activities, the locomotor functions of all experimental mice were examined after the behavioral tests described above. Placing, grasping, and righting reflex tests revealed no locomotor impairments in any of animals (Table 2). General behaviors, such as spontaneous activity and gait, were normal among the treatment groups. None of the animals displayed convulsions or hypermobility.

## Discussion

Despite several decades of intensive research on peripheral neuropathic pain, the mechanisms by which peripheral nerve injury precipitates nociceptive hypersensitivity remain incompletely understood. In this study, through employed comprehensive transcriptome profiling coupled with bioinformatic analyses. We identified a neuron-specific isoform of the RNA splicing regulator, Rbfox1, as a modulator of alternative RNA splicing in the DRG subsequent to peripheral nerve injury. Furthermore, we demonstrate that Rbfox1 plays a pivotal role in both the development of nociceptive hypersensitivity post-injury, primarily through its regulation of alternative RNA splicing of *Nrcam* in injured DRG neurons.

Although RNA splicing is a well-recognized contributor to transcriptome complexity and alternative RNA splicing are commonly observed among many neuronal genes during psychiatric and neurological disease, our knowledge regarding their underlying regulatory mechanisms and the functional impact is still very limited. Alternative RNA splicing is regulated by *cis*-regulatory enhancers and silencers located within pre-mRNAs interacting with *trans*-acting splicing factors. Several splicing factors, including Nova, Cwc22, SRSF1 have been implicated in development and maintenance of chronic pain [16,39,40]. Other splicing factors are also shown to play important roles in neuronal development and function, for example, PTBP1, CELF1, CUGBP1, and MBNL1 exhibit switched expression during brain development to regulate splicing of neuronal mRNAs [11,41,42]. However, RNA splicing factors are directly implicated in peripheral nerve injury-induced neuropathic pain in DRG have not been systematically characterized. In this study, using both unbiased bioinformatics analysis and experimental validations, we have uncovered a neuronal-specific form of RNA splicing regulator Rbfox1, which has a major contribution to peripheral nerve injury-induced neuropathic pain in primary sensory neurons of the DRG. Furthermore, we demonstrate that a skipped exon RNA splicing event for the exon 10 utilization of the *Nrcam* is a direct downstream target of Rbfox1 and has a critical role in the development of neuropathic pain. In summary, our study uncovered a previously uncharacterized Rbfox1/NrCAM regulatory circuit in injured DRGs in the context of neuropathic pain.

It has been reported that alternative RNA splicing affects several pain-related genes in the DRG, including genes encoding ion channels such as *Cacna1b*, which encodes the functional core of voltage-gated Ca_v_2.2 [43], and *SCN9A,* which encodes a subunit of the Na_v_1.7 channel [44]. Moreover, pain receptors, such as TRPV1 and TRPA1, also undergo splicing regulation [45,46]. NrCAM is a neuronal system cell-adhesion molecule of the L1 family of the immunoglobulin superfamily. It participates in neural development and some neurobiology disorders, such as neuropathic pain, drug addiction and ASD [32,47,48]. Our previous study showed that alternative RNA splicing of *Nrcam* exon 10 results in two splicing variants in the DRG and trigeminal ganglion: the long *Nrcam* variant (including exon 10) and the short *Nrcam* variant (excluding exon 10) [32]. *Nrcam* exon 10 encodes 19 amino acids located between immunoglobulin-like domains II and III (IgII and IgIII) of the NrCAM protein. There is a substantial change in the alternative RNA splicing of *Nrcam* exon 10 in injured DRG neurons of SNL mice. This change mainly includes exon 10 insertions in *Nrcam* mRNA, resulting in an increase in L-*Nrcam* variant expression. Correspondingly, the short *S-Nrcam* variant's expression decreases in injured DRG after SNL. Previous research has demonstrated that the binding of Rbfox1 upstream of an alternative exon inhibits its inclusion, whereas binding downstream facilitates such inclusion. In the context of the *Nrcam* gene, the Rbfox1 binding motif is positioned downstream of exon 10. An SNL-induced reduction in Rbfox1 expression would ostensibly lead to decreased exon inclusion. Nonetheless, post-SNL observations revealed an upregulation of the long *Nrcam* variant in DRG. This counterintuitive observation underscores the potential involvement of other splicing factors, RNA-binding proteins, and regulatory mechanisms in modulating *Nrcam* splicing. it's documented that lncRNA RUNX1-IT1, hnRNPH modulate the transcriptional expression of *Nrcam* [49,50].

There are three Rbfox paralogs in the mammalian nervous system, Rbfox1 is specifically expressed in neurons heart and muscle; while Rbfox2 is expressed in these tissues in addition to embryo, hematopoetic cells and embryonic stem cells (ESCs). Rbfox3 is expressed limited to neurons. All Rbfox proteins contain a single RNA recognition motif-type RNA binding domain that specifically binds the hexanucleotide (U)GCAUG. When this sequence is present in regulated exons or flanking introns, Rbfox is a splicing regulator that promotes or represses exon inclusion [51]. Studies in knockout mice revealed the role of Rbfox1 in regulating neuronal splicing networks involved in neuronal excitability [24]. Multiple vertebrate species, including mice, rats, and humans, contain the Rbfox1 RRM near the targeted *Nrcam* exon 10, indicating that the Rbfox1/NrCAM regulatory pathway is a highly conserved mechanism in transcriptome regulation. Rbfox1 expression was dramatically decreased in injured DRG, but not intact DRGs and ipsilateral spinal cord, in preclinical mouse models of neuropathic pain caused by SNL or CCI. Both models are complementary, as SNL-induced pain results mainly from direct nerve injury (28), whereas CCI induced pain is initiated primarily by ischemia. The decreased levels of Rbfox1 occurred predominantly in small and medium DRG neurons, although it's expressed in all types of DRG neurons. Interestingly, peripheral tissue injury and inflammation did not substantially change Rbfox2 expression, while prior research has indicated a mutual compensatory relationship between the Rbfox1 and Rbfox2 [51].

NrCAM palys a pivotal role in neural development, with functions encompassing synapse formation, cell proliferation and differentiation, axon guidance and growth, and myelinate nerve structure formation [52]. Beyond its development roles, NrCAM has been linked to drug addiction and certain psychiatric disorders, notably autism [47,53]. Our results indicate that alternative RNA splicing of *Nrcam* exon 10 in DRG contributes to Rbfox1-mediated neuropathic pain resulting from peripheral nerve injuries. While the molecular mechanisms for these varied functions are still under exploitation, it's notable that NrCAM exerts its oncogenic activity involves binding to epidermal growth factor receptor (EGFR). Activation of EGFR in DRG is implicated in chronic pain induction, and inhibiting EGFR has been observed to alleviated pain hypersensitivities caused by chronic compression of DRG [54]. Furthermore, the downstream EGFR cellular pathway, PI3K/Akt/mTOR, plays a role in neuropathic pain progression [55]. It is possible that the effects of *Nrcam* variants on neuropathic pain arise from EGFR-triggered activation of the intracellular MAPK/ERK and PI3K/Akt/mTOR pathways. Further studies are essential to validate this hypothesis. An acknowledged limitation of our study is the omission of investigations into the potential effects of altered *Nrcam* splicing isoforms on DRG neuron excitability.

In conclusion, our study shows that the gain or loss of Rbfox1 function regulates nerve injury-induced neuropathic pain. We identify NrCAM as a significant Rbfox1 target in injured DRGs after peripheral nerve injury. Consequently, Rbfox1-mediated RNA splicing can be a potential novel target intervention against pathological conditions, and restoring Rbfox1 abundance and function can be a new therapeutic approach for peripheral nerve injury-induced neuropathic pain.

## Author contributions

J.-J. Yang, Y.-Q. Ai, and L. He conceived the project and designed the experiments. J.-J. Yang and Y.-Q. Ai coordinated and supervised all experiments. L. He, H.-Y. Guo, K.-C. Zhu, D. Li, and C.-F. Zhang conducted most molecular, biochemical, and morphological experiments. L. He, H.-Y. Guo, C.-F. Zhang, and H.-W. Wang performed most of the animal surgery and behavioral experiments. L. He, H.-Y. Guo, Y.-Q. Ai, and J.-J. Yang analyzed the data. L. He and H.-W. Wang did the statistical analysis. L. He wrote a draft of the manuscript. L. He, Y.-Q Ai, and J.-J. Yang edited the final manuscript. All authors read and discussed the manuscript.

## Data availability statement

All data associated with this study are present in the manuscript or the Supporting Information.

## Declaration of competing interest

The authors declare that they have no known conflicting financial interests or personal relationships that could have appeared to influence the work reported in this paper.
