# Supplementary material for: Rbfox1 regulates alternative splicing of Nrcam in primary sensory neurons to mediate peripheral nerve injury-induced neuropathic pain
Source: Neurotherapeutics. 2023 Dec 19;21(1):e00309. doi: 10.1016/j.neurot.2023.e00309 (PMC10903086; doi:10.1016/j.neurot.2023.e00309)
Supplement: Multimedia component 3 [file mmc3.pptx]

## Slide 1
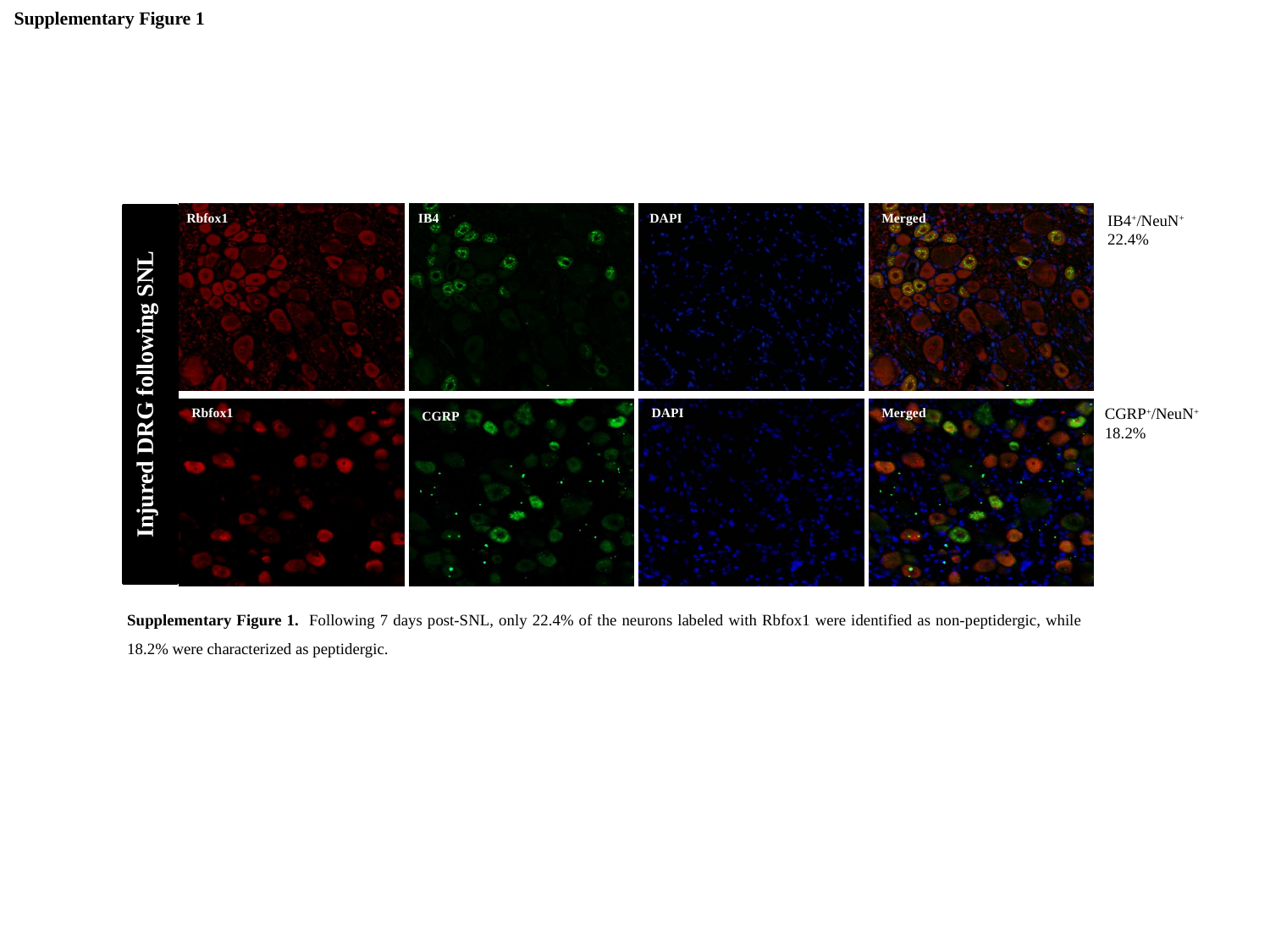

Supplementary Figure 1
Rbfox1
IB4
DAPI
Merged
Injured DRG following SNL
Rbfox1
DAPI
Merged
CGRP
IB4+/NeuN+
22.4%
CGRP+/NeuN+
18.2%
Supplementary Figure 1. Following 7 days post-SNL, only 22.4% of the neurons labeled with Rbfox1 were identified as non-peptidergic, while 18.2% were characterized as peptidergic.

## Slide 2
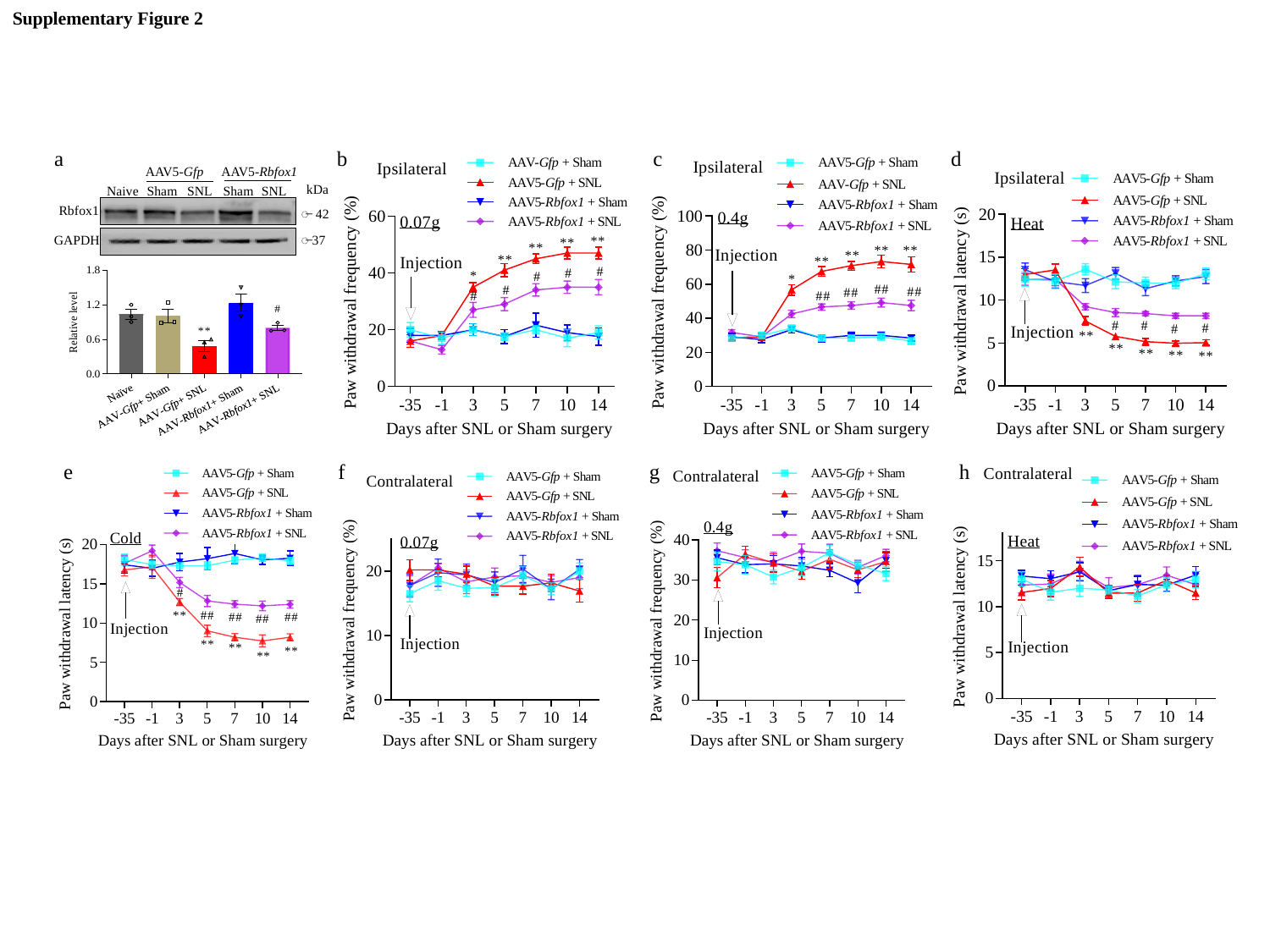

Supplementary Figure 2
a
b
c
d
AAV5-Gfp
AAV5-Rbfox1
kDa
Naive
Sham
SNL
Sham
SNL
Rbfox1
̶ 42
̶ 37
GAPDH
e
f
g
h

## Slide 3
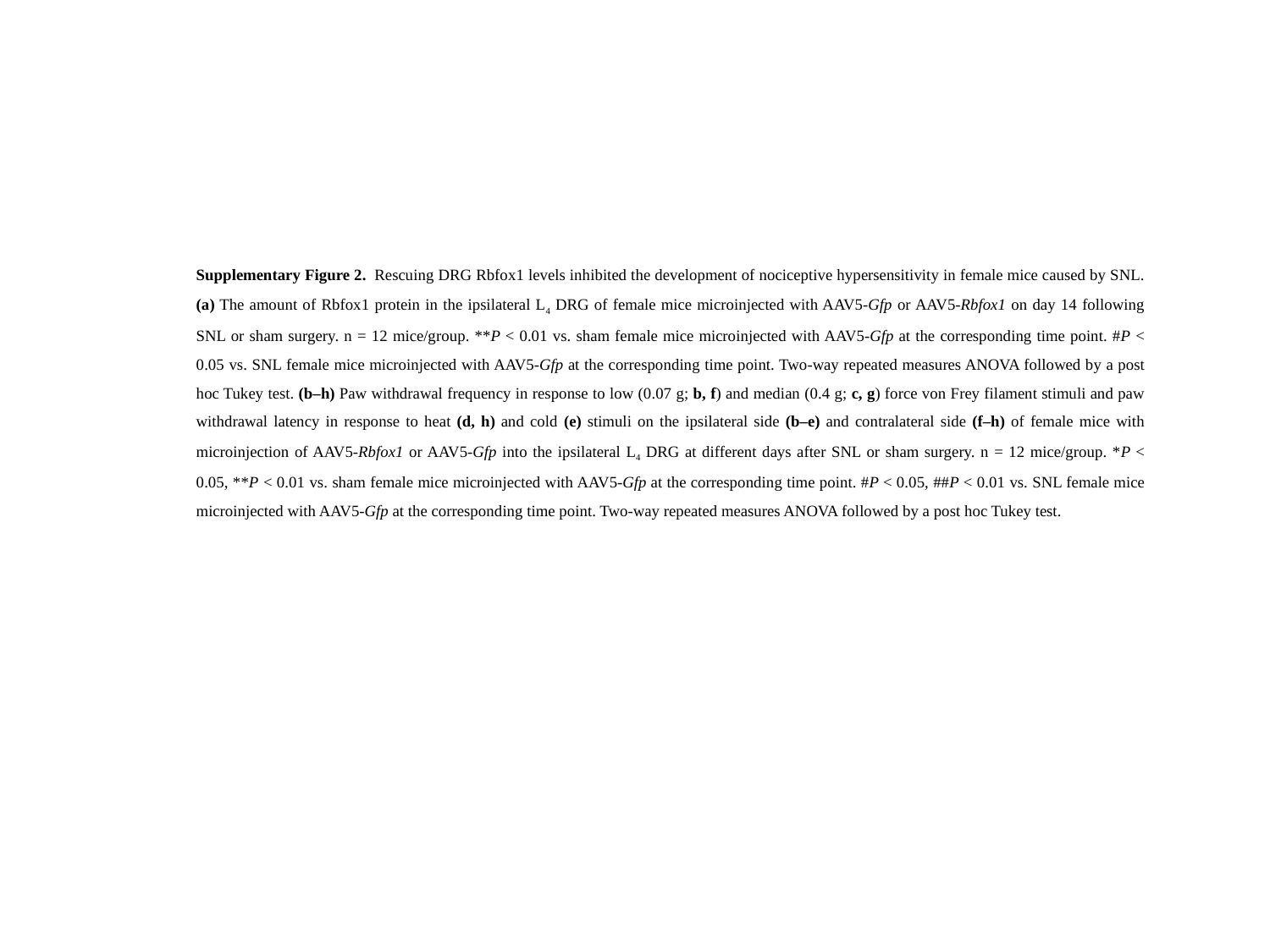

Supplementary Figure 2. Rescuing DRG Rbfox1 levels inhibited the development of nociceptive hypersensitivity in female mice caused by SNL. (a) The amount of Rbfox1 protein in the ipsilateral L4 DRG of female mice microinjected with AAV5-Gfp or AAV5-Rbfox1 on day 14 following SNL or sham surgery. n = 12 mice/group. **P < 0.01 vs. sham female mice microinjected with AAV5-Gfp at the corresponding time point. #P < 0.05 vs. SNL female mice microinjected with AAV5-Gfp at the corresponding time point. Two-way repeated measures ANOVA followed by a post hoc Tukey test. (b–h) Paw withdrawal frequency in response to low (0.07 g; b, f) and median (0.4 g; c, g) force von Frey filament stimuli and paw withdrawal latency in response to heat (d, h) and cold (e) stimuli on the ipsilateral side (b–e) and contralateral side (f–h) of female mice with microinjection of AAV5-Rbfox1 or AAV5-Gfp into the ipsilateral L4 DRG at different days after SNL or sham surgery. n = 12 mice/group. *P < 0.05, **P < 0.01 vs. sham female mice microinjected with AAV5-Gfp at the corresponding time point. #P < 0.05, ##P < 0.01 vs. SNL female mice microinjected with AAV5-Gfp at the corresponding time point. Two-way repeated measures ANOVA followed by a post hoc Tukey test.

## Slide 4
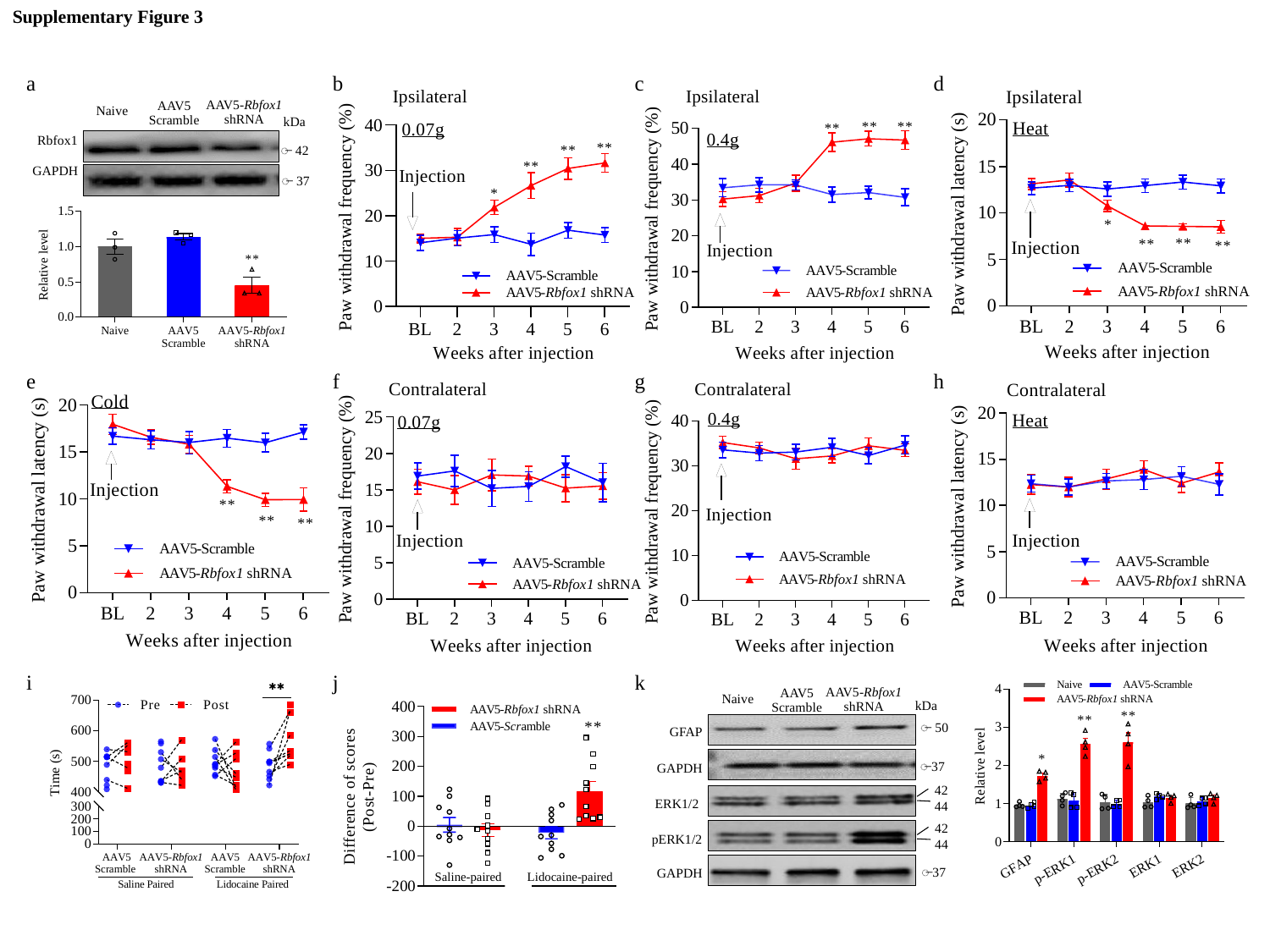

Supplementary Figure 3
a
b
c
d
AAV5-Rbfox1 shRNA
AAV5
Scramble
Naive
kDa
Rbfox1
̶ 42
GAPDH
̶ 37
e
f
g
h
i
j
k
AAV5-Rbfox1
shRNA
AAV5
Scramble
Naive
kDa
̶ 50
GFAP
̶ 37
GAPDH
42
44
ERK1/2
42
44
pERK1/2
̶ 37
GAPDH

## Slide 5
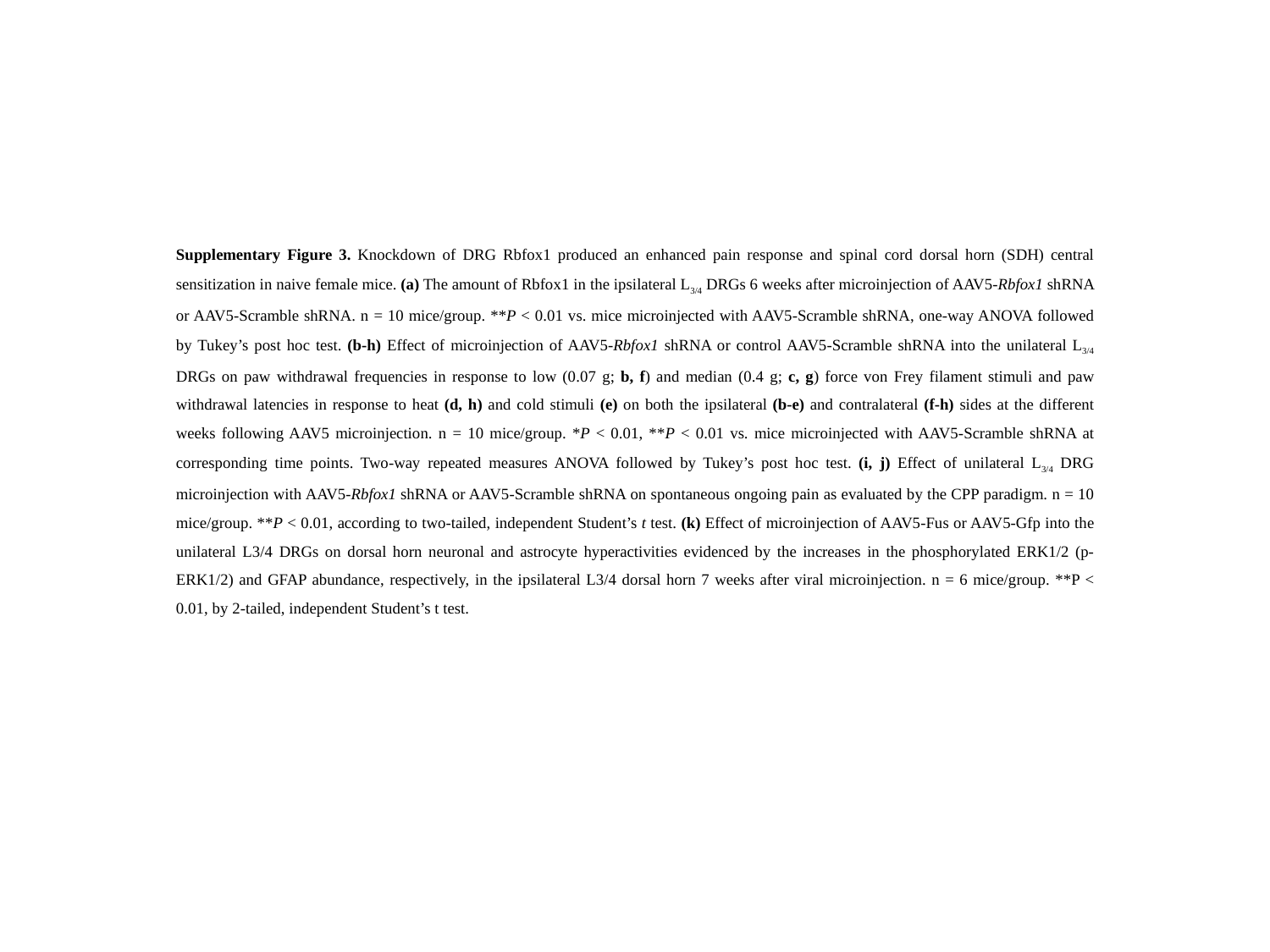

Supplementary Figure 3. Knockdown of DRG Rbfox1 produced an enhanced pain response and spinal cord dorsal horn (SDH) central sensitization in naive female mice. (a) The amount of Rbfox1 in the ipsilateral L3/4 DRGs 6 weeks after microinjection of AAV5-Rbfox1 shRNA or AAV5-Scramble shRNA. n = 10 mice/group. **P < 0.01 vs. mice microinjected with AAV5-Scramble shRNA, one-way ANOVA followed by Tukey’s post hoc test. (b-h) Effect of microinjection of AAV5-Rbfox1 shRNA or control AAV5-Scramble shRNA into the unilateral L3/4 DRGs on paw withdrawal frequencies in response to low (0.07 g; b, f) and median (0.4 g; c, g) force von Frey filament stimuli and paw withdrawal latencies in response to heat (d, h) and cold stimuli (e) on both the ipsilateral (b-e) and contralateral (f-h) sides at the different weeks following AAV5 microinjection. n = 10 mice/group. *P < 0.01, **P < 0.01 vs. mice microinjected with AAV5-Scramble shRNA at corresponding time points. Two-way repeated measures ANOVA followed by Tukey’s post hoc test. (i, j) Effect of unilateral L3/4 DRG microinjection with AAV5-Rbfox1 shRNA or AAV5-Scramble shRNA on spontaneous ongoing pain as evaluated by the CPP paradigm. n = 10 mice/group. **P < 0.01, according to two-tailed, independent Student’s t test. (k) Effect of microinjection of AAV5-Fus or AAV5-Gfp into the unilateral L3/4 DRGs on dorsal horn neuronal and astrocyte hyperactivities evidenced by the increases in the phosphorylated ERK1/2 (p-ERK1/2) and GFAP abundance, respectively, in the ipsilateral L3/4 dorsal horn 7 weeks after viral microinjection. n = 6 mice/group. **P < 0.01, by 2-tailed, independent Student’s t test.
